# Solar Radiation Induces Non-Nuclear Perturbations and a False Start to Regulated Exocytosis in *Cryptosporidium parvum*

**DOI:** 10.1371/journal.pone.0011773

**Published:** 2010-07-23

**Authors:** Brendon J. King, Daniel Hoefel, Pao Ee Wong, Paul T. Monis

**Affiliations:** 1 South Australian Water Corporation, Australian Water Quality Centre, Cooperative Research Centre for Water Quality and Treatment, Adelaide, South Australia, Australia; 2 Department of Medical Biotechnology, School of Medicine, Flinders University, Adelaide, South Australia, Australia; BMSI-A*STAR, Singapore

## Abstract

Stratospheric ozone depletion, climate warming and acidification of aquatic ecosystems have resulted in elevated levels of solar radiation reaching many aquatic environments with an increased deleterious impact on a wide range of living organisms. While detrimental effects on living organisms are thought to occur primarily through DNA damage, solar UV can also damage cellular proteins, lipids and signalling pathways. *Cryptosporidium*, a member of the eukaryotic phylum Apicomplexa, contain numerous vesicular secretory organelles and their discharge via regulated exocytosis is essential for the successful establishment of infection. Using flow cytometric techniques we demonstrate that solar UV rapidly induces sporozoite exocytosis resulting in a significant reduction in the ability of sporozoites to attach and invade host cells. We found that solar UV induced sporozoite membrane depolarization, resulting in reduced cellular ATP and increased cytosolic calcium. These changes were accompanied by a reduction in the internal granularity of sporozoites, indicative of apical organelle discharge, which was confirmed by analysis of sporozoites with an exocytosis-sensitive dye. The precise timing of apical organelle discharge in the presence of a compatible host cell is critical for sporozoite attachment and invasion. Our results demonstrate for the first time how solar UV radiation can interfere with exocytosis, a fundamental cellular process in all eukaryotic cells. We contend that not only may the forecast increases in solar radiation in both aquatic and terrestrial environments significantly affect members of the Apicomplexa, solar UV-induced membrane depolarizations resulting in cytosolic calcium perturbation may affect a wider range of eukaryotic organisms through antagonistic effects on a myriad of calcium dependant cellular functions.

## Introduction 

The eukaryotic phylum Apicomplexa comprises more than 5000 species of pathogenic protozoa, members of which cause considerable morbidity and mortality in humans, livestock and wildlife [1]. Within this phylum the largest group of parasites, the coccidians, maintain their lifecycle by shedding infective oocysts within the host faeces, with the aquatic environment serving as an excellent vehicle for transmission and survival of this stage. Massive loss of stratospheric ozone during the past two decades, accompanied by acid deposition and climate warming, has resulted in marked increases in exposure of the upper water column to solar ultraviolet radiation [2]–[5]. While UV exposure has been identified as detrimental to a wide range of organisms, scant attention has been paid to its affect on parasites in aquatic systems.

Protozoan parasites of the Apicomplexan genus *Cryptosporidium* are ubiquitous and a significant enteropathogen of a wide range of vertebrates [6]. It is well established that the infectious form, the oocyst, is environmentally robust and capable of persisting in the environment for extended periods [7]. Recent research however, has highlighted the vulnerability of *Cryptosporidium parvum* to solar UV [8]–[10]. Although short wave UV radiation can disturb most macro-molecules, studies in animal systems suggest that damage to the structure and function of DNA is the primary mechanism responsible for cell injury and loss of viability [11]. Cyclobutane pyrimidine dimers (CPDs) are the major aberrant DNA photoproduct induced by solar UV [12], making up approximately 75% of all UV-induced photoproducts, and their accumulation in populations has been shown to be highly toxic and mutagenic [13].

However, the biological effects of solar UV radiation have been shown to be diverse, including inhibition of motility and orientation, protein destruction, pigment bleaching and photoinhibition of photosynthesis [14]–[17]. *Cryptosporidium*, along with other members of the Apicomplexa, share common apical secretory apparatus essential for locomotion, attachment and cellular invasion [18]. The regulated discharge of these organelles is essential for successful host cell invasion by a number of these parasites [18], [19], with any disruption having potentially dire consequences for the successful establishment of infection.

We undertook outdoor microcosm studies to investigate the formation of CPDs in *C. parvum* oocysts and relate CPD formation to reductions in oocyst infectivity, in order to determine whether *Cryptosporidium* susceptibility resulted from a high load of DNA lesions. However, we were unable to detect extensive CPD formation and DNA damage in oocysts exposed solar UV. To investigate whether other cellular targets may be responsible for *C. parvum's* hypersensitivity to solar UV, we undertook a further series of outdoor microcosm experiments aimed at relating the reductions witnessed in oocyst infectivity induced by solar radiation with parameters relating to regulated exocytosis. We demonstrate for the first time that solar UV radiation can interfere in exocytosis, a fundamental cellular process in all eukaryotic cells.

## Results and Discussion

### Quantification of DNA damage induced by UV-C and solar radiation

We undertook outdoor microcosm studies to investigate the formation of CPDs in *C. parvum* oocysts and relate CPD formation to reductions witnessed in oocyst infectivity. These experiments were performed to determine whether *Cryptosporidium* susceptibility resulted from a high load of DNA lesions. Immunoassays have been widely used for quantification of solar UV-induced damage in a diverse range of organisms including bacteria, phytoplankton, plants and animals [13], [20]–[22]. More recently, quantitative PCR has been described as a suitable tool for analysis of nuclear DNA damage in ecotoxicologic studies [23], [24]. Consequently, we employed immunoblot and quantitative sequence detection (QSD) assays to quantify UV-induced DNA damage.

**Figure 1:**
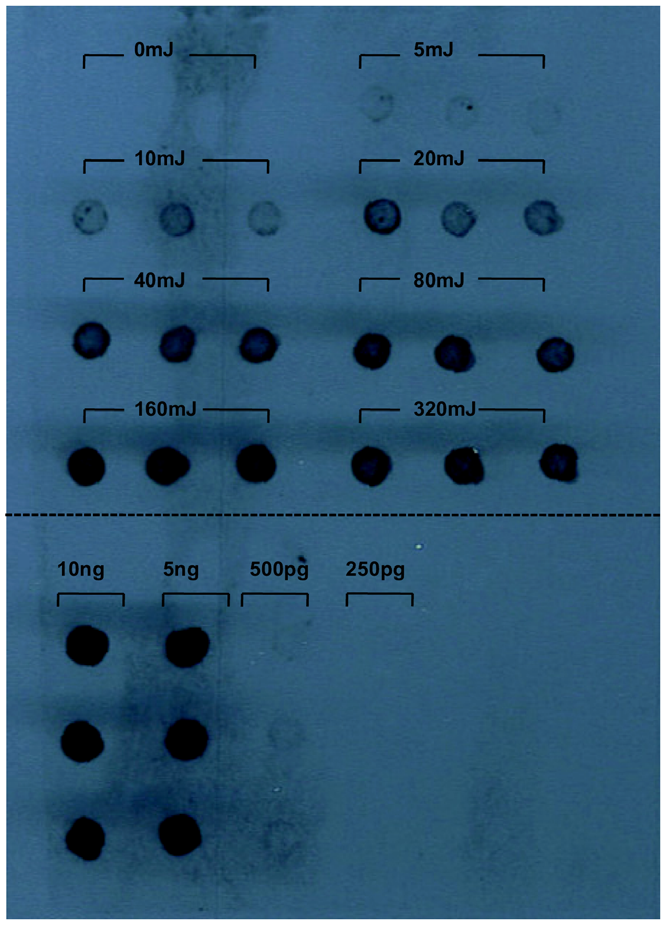
Quantification of the effect of UV-C on Cyclobutane dimer formation within *Cryptosporidium* oocysts. DNA extracts of oocysts exposed to a variety of UV-C dosages using a collimated beam were spotted and fixed to a Nylon Hybond^+^ membrane in triplicate. UV-C induced damage was assessed through the use of a monoclonal antibody that recognized and bound specifically to cyclobutane pyrimidine dimmers (CPDs). The chemiluminescent treated blot was exposed to photographic film for 2 minutes. Plasmid DNA exposed to 360 mJ/cm^2^ of UV-C was used as a standard (250pg-10ng) and is located in the lower panel beneath the dashed line.

**Figure 2:**
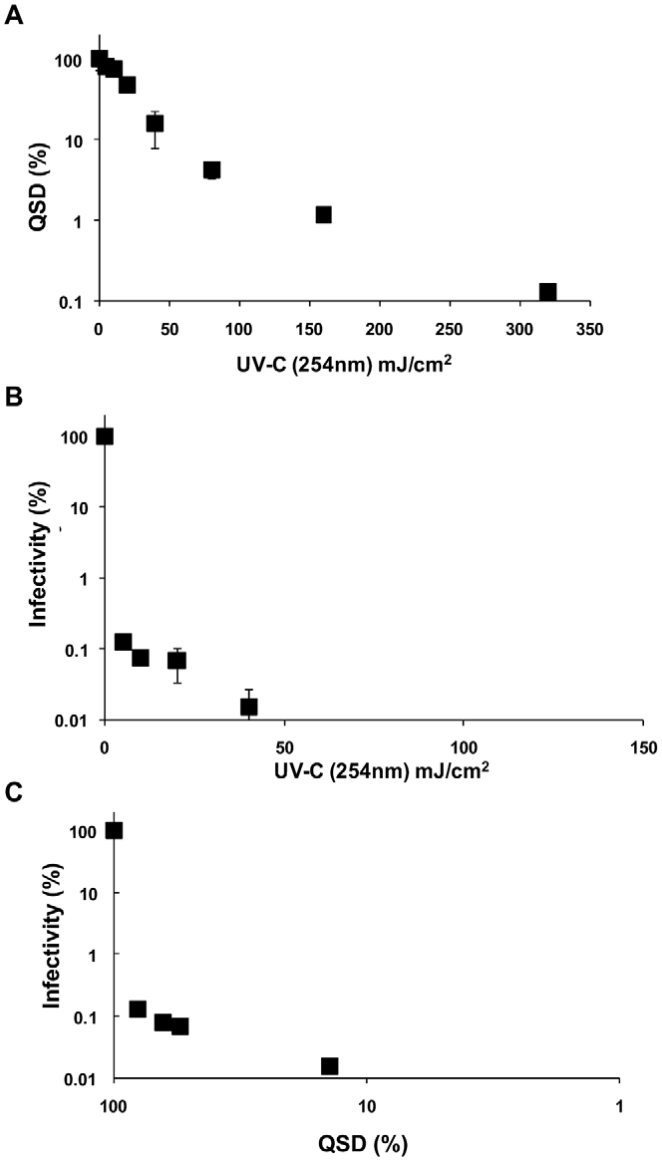
Quantification of the effect of UV-C on DNA damage within *Cryptosporidium* oocysts using QSD. **A**) DNA extracts of oocysts exposed to a variety of UV-C dosages using a collimated beam were amplified using a TaqMan PCR assay. Non-irradiated oocysts were used as controls, and the treatments calculated as a percentage of the control. Significant differences (*t*-test, P<0.05) were evident with UV-C dosages of 10 mJ/cm^2^ or greater. **B**) Oocyst infectivity was determined using a cell culture TaqMan PCR infectivity assay for the different UV exposures. Non-irradiated oocysts were used as controls, and the treatments calculated as a percentage of the control. **C**) A relationship was able to be established between QSD inhibition and reductions witnessed in oocyst infectivity. Error bars indicate standard deviations for infectivity (n = 3) and QSD (n = 3).

At present UV-C does not reach the terrestrial surface, however it is extremely effective in the induction of DNA photoproducts [25]. Therefore, UV-light using a collimated beam apparatus with a low pressure lamp which emitted monochromatic radiation with a peak at 254nm was used to assess the suitability and sensitivity of both detection assays for quantifying DNA damage. A cell-culture TaqMan PCR assay was used to quantify oocyst inactivation thus enabling the establishment of a relationship between DNA damage and *Cryptosporidium* oocyst inactivation for UV-C light. Both immunoblot and QSD assays were identified as suitable methods for detecting DNA lesions in irradiated oocysts inactivated to varying degrees over a range of UV-C dosages (Figs. 1, 2). The immunoblot assay was determined to be the most sensitive and able to detect CPDs following a dose of 5mJ/cm^2^ and greater after a 2 minute photographic exposure of the chemiluminescent treated blot (Fig. 1). Control experiments (0mJ/cm^2^) demonstrated that the antibody did not detectably bind to oocyst DNA that had not been exposed to UV light. Exposure to UV-C light also resulted in an inability of the damaged DNA to serve as a PCR template with significant differences (*t*-test, P<0.05) evident with UV-C dosages of 10 mJ/cm^2^ and greater (Fig. 2). We hypothesized that we should be able to readily quantify DNA lesions for similar reductions in oocyst infectivity induced by solar insolation if the load of lesions responsible for oocyst inactivation was comparable.

**Figure 3:**
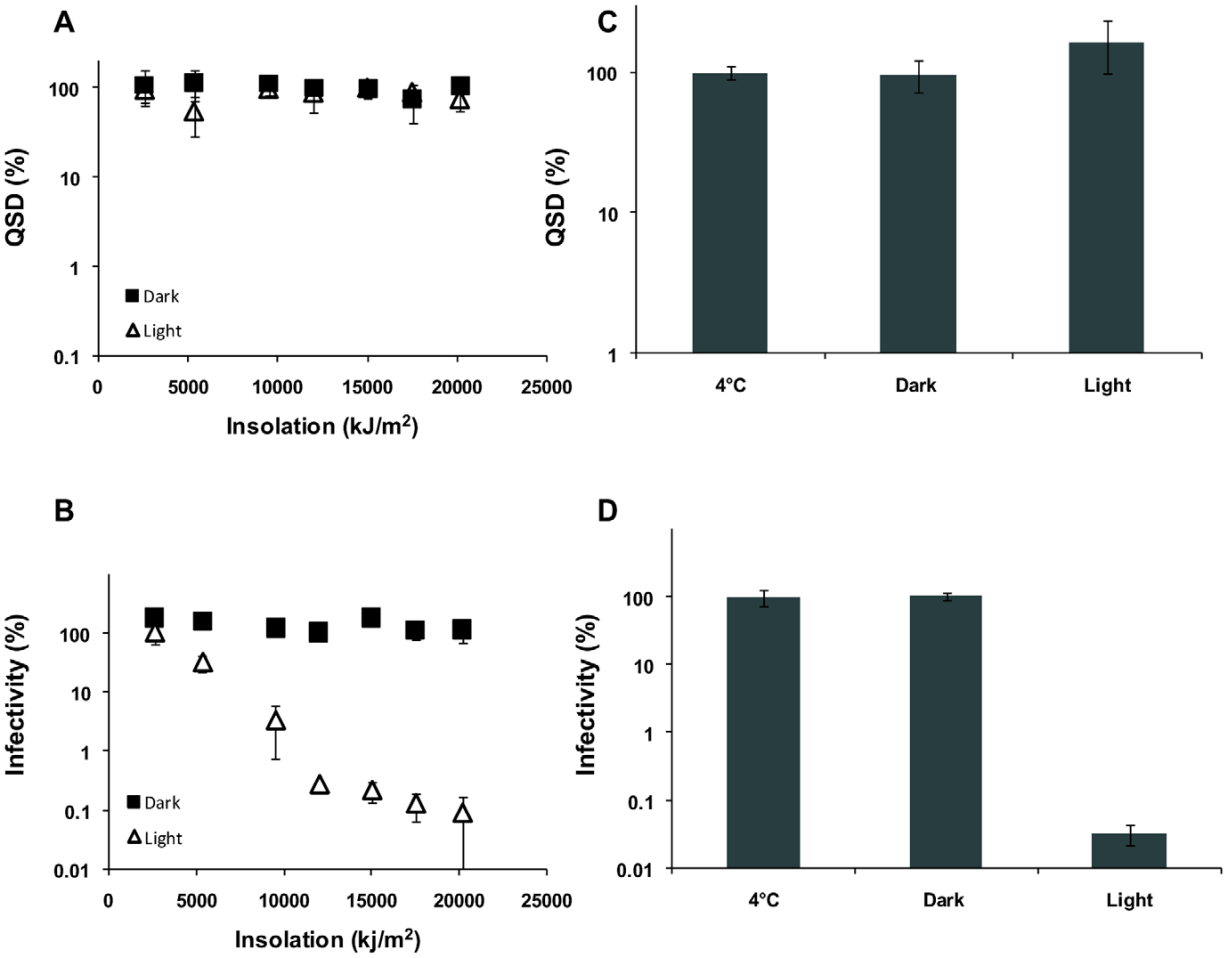
Quantification of the effect of solar insolation on DNA damage within *Cryptosporidium* oocysts using QSD. **A**) DNA extracts of oocysts exposed to a variety of solar insolation levels for both light and dark treatments on a clear sky day with a UV maximum of 4 were amplified using a TaqMan PCR assay. Non-irradiated oocysts kept at 4°C were used as controls, and treatments calculated as a percentage of the control. **B**) Oocyst infectivity was determined using a cell culture TaqMan PCR infectivity assay for the different solar insolation treatments and the infectivity of the treatments calculated as a percentage of the 4°C control. **C**) DNA extracts of oocysts exposed to a higher level of solar insolation (37,208kJ) for both light and dark treatments in an outdoor microcosm experiment performed over two consecutive days, with solar UV index maxima of 4 (clear sky day) and 2 (cloudy) respectively, were amplified using a TaqMan PCR assay. **D**) Oocyst infectivity was determined using a cell culture TaqMan PCR infectivity assay for this higher level of solar insolation (37,208kJ) and the infectivity of the treatments calculated as a percentage of the 4°C control. Error bars indicate standard deviations for infectivity (n = 3) and QSD (n = 3).

Outdoor microcosm experiments were therefore undertaken to investigate the formation of DNA lesions induced by solar insolation and to determine if the relationship between DNA damage and oocyst inactivation induced by solar insolation was comparable to the relationship quantified for UV-C light. However, while CPD formation and DNA damage was detected in oocysts exposed to UV-C (254nm), we were unable to readily detect CPD formation or DNA damage in oocysts exhibiting similar or greater levels of inactivation by solar UV (Fig. 3) (Figs. S1, S2). DNA damage could not be detected in solar irradiated oocysts using QSD even when greater than a 3 log reduction in infectivity was achieved (Fig. 3C, D). While similar reductions in oocyst infectivity from UV-C resulted in a reduction in QSD ranging from 45% to 86% of the non-irradiated controls (Fig. 2C).

The level of CPDs detected in solar irradiated oocysts was close to the limit of detection for the immunoblot assay. Increasing photographic film exposure from 2 to 40 minutes for the chemiluminescent treated blot failed to detect any CPDs in the solar insolation treatments (Fig. S1), including those treatments which achieved equivalent to a 3 log reduction in oocyst infectivity (Fig. 3B). However, CPDs could readily be detected in oocysts inactivated to similar degrees from UV-C exposure after photographic film exposure of the treated blot for only 2 minutes (Fig. 1). CPDs were able to be detected in solar irradiated oocysts, but only when the amount of oocyst DNA blotted was increased by 10 fold, exposure to photographic film extended to 45 minutes and greater than a 3 log reduction in oocyst infectivity achieved (Fig. S2). While it appears that *Cryptosporidium* susceptibility to solar radiation is not resultant from a high load of DNA lesions, we cannot rule out the possibility that a low load of CPDs within sporozoites may still considerably contribute to the reductions witnessed in infectivity.

It is noteworthy that CPDs have been identified as able to be repaired in oocysts after UV-C irradiation [26], [27] and all the genes for the major components of a nucleotide excision repair complex have been identified in *C. parvum* [28]. While it has been demonstrated that oocysts have the potential to repair UV-C induced damage, oocyst reactivation has not been demonstrated to occur under conditions examined, and it has been suggested that other components such as proteins needed for infection may be irreversibly damaged [20]. Our inability to measure extensive DNA damage in solar irradiated inactivated oocysts lead us to investigate other cellular targets that may be responsible for *C. parvum* susceptibility to solar irradiation.

### Quantification of solar induced non-nuclear changes

To investigate whether other cellular targets may be responsible for *C. parvum's* hypersensitivity to solar UV we undertook a further series of outdoor microcosm experiments. Global solar radiation was measured onsite using a pyranometer and the daily UV index recorded from the Australian Government Bureau of Meteorology website. The UV index was used to calculate the expected T_90_ value (time taken to achieve 90% oocyst inactivation), and microcosms sampled near this time and a second time-point of twice the duration to achieve desired levels of inactivation on days of varying solar radiation levels (Fig. S3 and Table S1). Oocyst infectivity was determined using a cell-culture TaqMan assay (Fig. S4) and *Cryptosporidium* sporozoites released following oocyst excystation were stained with the membrane potential sensitive dye DiBAC_4_(3) before flow cytometric analysis to quantify changes to sporozoite membrane polarization.

**Figure 4:**
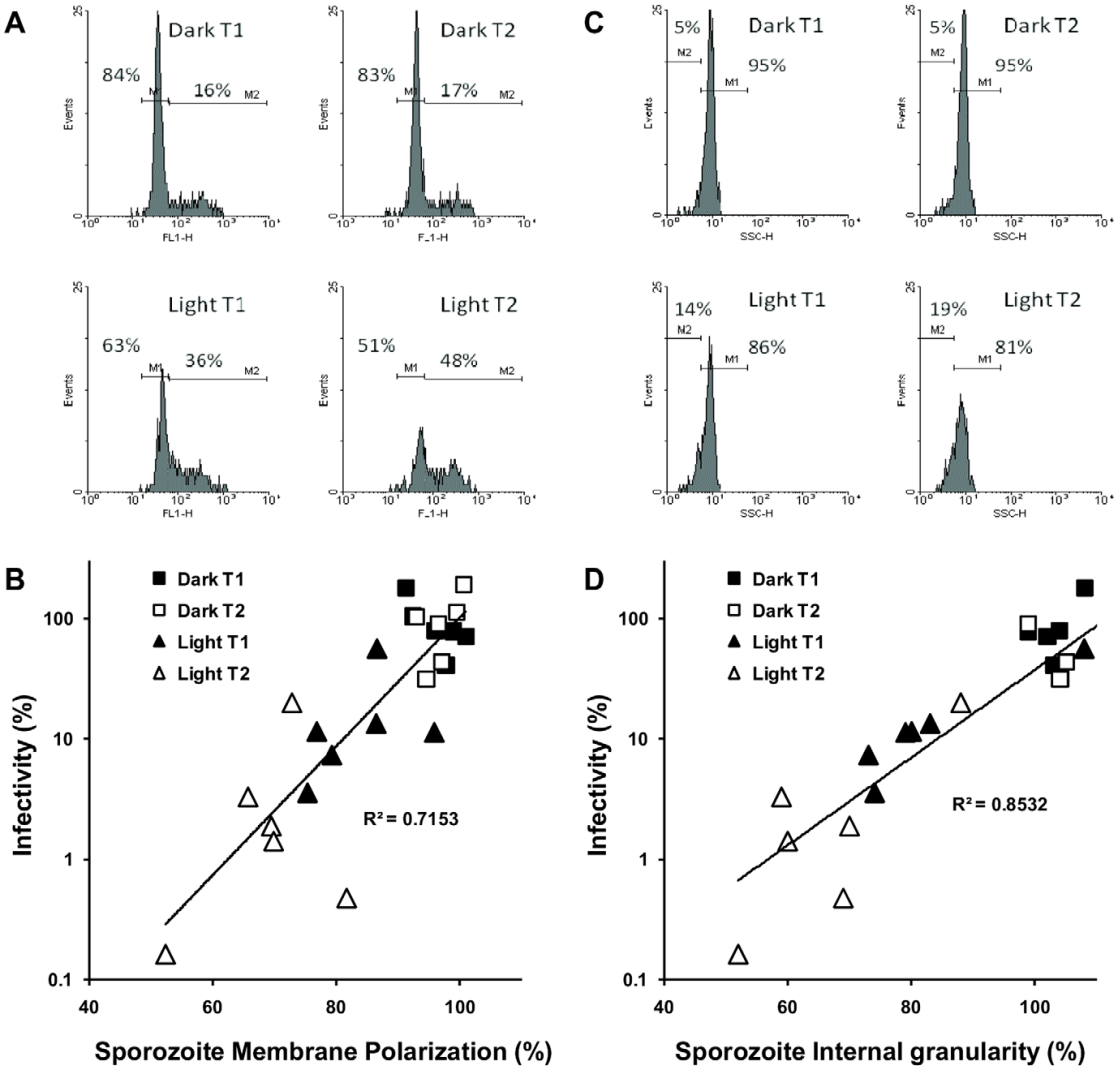
The effect of solar insolation on sporozoite membrane polarization, internal granularity and infectivity. *Cryptosporidium* oocysts exposed to variable solar insolation conditions in six independent outdoor microcosm experiments were excysted and sporozoites stained with the membrane potential sensitive dye DiBAC_4_(3) before flow cytometric analysis. Oocyst microcosms were sampled at two levels of insolation (T1 and T2) for each microcosm experiment (see Table S1). **A**) Representative histograms of the fluorescence intensity (FL-1) of excysted sporozoites from dark and light irradiated oocysts of a single microcosm experiment demonstrate large changes in sporozoite membrane polarization for light irradiated oocysts. **B**) Under varying solar UV indices a strong correlation was identified between reductions in oocyst infectivity and reductions in sporozoite membrane potential for all six microcosm experiments. Non-irradiated oocysts were used as controls, and the treatments calculated as a percentage of the control. **C**) Representative histograms of the Side Scatter Channel (SSC) of excysted sporozoites from dark and light irradiated oocysts of a single microcosm experiment demonstrate changes in the internal granularity for the light irradiated oocysts. **D**) Under varying solar UV indices a strong correlation was identified between reductions in oocyst infectivity and reductions in sporozoite internal granularity for all six microcosm experiments. Non-irradiated oocysts were used as controls, and the treatments calculated as a percentage of the control.

DiBAC_4_(3) is a lipophilic and anionic bis-oxonal membrane potential dye with uptake of the dye restricted to depolarized cells or cells with disrupted cytoplasmic membranes. The fluorescent dye accumulates inside the cells by binding to intracellular membranes and proteins, increasing the green fluorescent intensity of the cell [29]. Flow cytometric analysis revealed a significant depolarization of the sporozoite membrane of oocysts exposed to solar UV radiation in comparison to that of the dark controls, with increased UV dosage resulting in further membrane depolarization (Fig. 4A) (Fig. S5). A strong correlation (r^2^ = 0.72) was identified between oocyst infectivity and sporozoite membrane polarization across a range of solar irradiances, with decreases in membrane polarization accompanied by exponential decreases in infectivity (Fig. 4B).

Depolarization-dependent rises in cytoplasmic Ca^2+^ have been shown to trigger exocytosis and the release of proteins in a number of eukaryotic systems [30]–[32]. Interestingly, flow cytometric analysis of the DiBAC_4_(3) stained sporozoites on the side scatter channel (SSC) identified significant decreases in the internal granularity of sporozoites exposed to solar UV radiation, an indication of exocytosis, with increased solar UV dosage resulting in larger reductions in internal granularity (Fig. 4C) ( Fig. S6). An even stronger correlation (r^2^ = 0.85) across the same range of solar irradiances was identified between sporozoite internal granularity and infectivity, with decreased internal granularity accompanied by exponential decreases in infectivity (Fig. 4D). SSC is a function of a number of parameters including the intracellular refractive index, the complexity of the intracellular organelles and their reflective properties, and, in particular, cell granularity [33]. It has been previously demonstrated that decreased cell granularity is linked to secretion, an indication of the involvement of exocytosis [34]. When cells have undergone exocytosis, their refractility is lost and their ability to scatter light at 90° is correspondingly diminished [35], [36].

*Cryptosporidium* sporozoites contain a single rhoptry, numerous micronemes and several dense granules predominantly localised at the apical region of the sporozoite contributing to a complex internal granularity [37]. Along with other members of the Apicomplexa the regulated discharge of these organelles is essential for successful host cell invasion [18], [19]. *C. parvum* sporozoites have previously been demonstrated capable of discharging these organelles in the absence of host cells [38], which can be detected cytometrically by decreased SSC of the sporozoite population accompanied by a rapid depolarization of the sporozoite membrane [39]. We postulated that these changes in sporozoite internal granularity and membrane polarization were strongly suggestive of an early discharge of apical organelles within sporozoites. Additionally, a preliminary outdoor microcosm experiment performed on a single day with more intensive sampling across a wider range of solar insolations identified even stronger correlations between reductions in oocysts infectivity, and decreased internal sporozoite granularity and membrane polarization (Fig. S7).

Further to this, we evaluated whether solar UV may induce apical organelle discharge by way of membrane depolarization, leading to an accelerated run-down of intracellular ATP, failure of ionic pumps, a subsequent inability to clear cytosolic calcium and premature activation of the secretory system. To confirm this we analyzed both intracellular ATP levels of excysted sporozoites and oocysts and quantified cytosolic calcium levels using the calcium indicator Fluo-4 AM ester.

**Figure 5:**
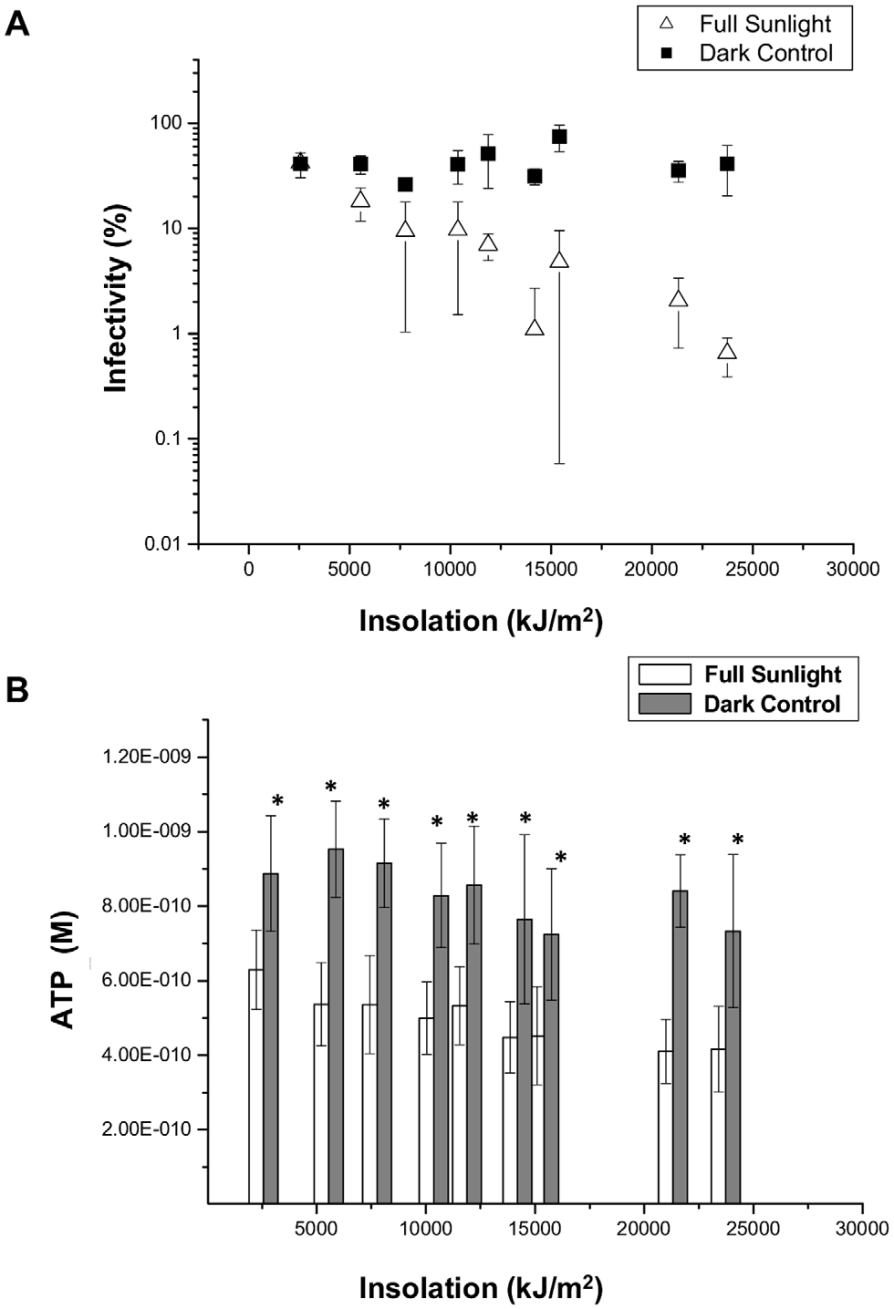
The effect of solar insolation on oocyst infectivity and ATP content during a microcosm experiment. A microcosm experiment was undertaken exposing *Cryptosporidium* oocysts to solar radiation with intensive sampling. The experiment was performed over two consecutive clear sky days, with solar UV index maxima of 3 for both days. **A**) Oocyst infectivity was determined using a cell culture TaqMan PCR infectivity assay for both dark and light irradiated oocysts. Non-irradiated oocysts kept at 4°C were used as controls, and treatments calculated as a percentage of the control. **B**) Oocysts sampled at the same time-points corresponding to each insolation level were incubated at 37°C for a 24 hour holding period before ATP extraction and analysis. ATP concentration of oocysts is expressed as molarity (M). An asterisk above a pair of bars indicate statistically significant effects (*t*-test, P<0.05). Error bars indicate standard deviations (n = 3).

When excysted, sporozoites consume ATP rapidly due to the energy intensive nature of helical gliding and a finite energy reserve [39], [40]. Over a range of solar irradiances, ATP levels of intact oocysts and excysted sporozoites from solar irradiated treatments showed a more rapid decrease than those from the dark controls (Fig. 5) (Figs. S8, S9). Oocysts have limited internal energy resources and are unable to maintain infectivity and ATP levels for prolonged periods at high metabolic temperatures. Sporozoite membrane depolarization induced by solar radiation resulted in an accelerated depletion of cellular ATP. This is most likely due to increased activity of ATP dependant ionic pumps trying to maintain ionic potential, which was further exacerbated by increasing the holding period at a higher metabolic temperature (Fig. S9).

**Figure 6:**
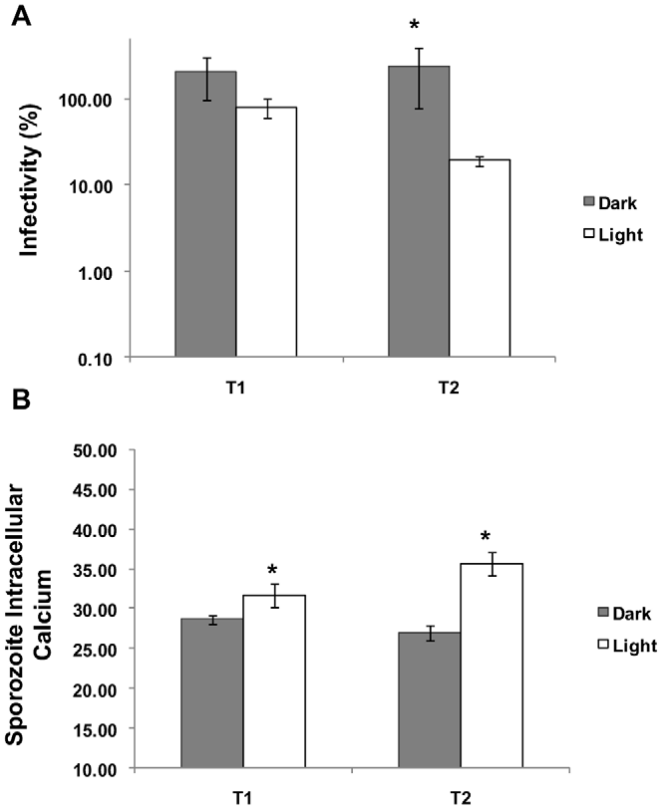
The effect of solar insolation on *Cryptosporidium* oocyst infectivity and sporozoite intracellular calcium. A microcosm experiment was performed on a clear sky day with a solar UV index maximum of 11 in order to investigate changes oocyst infectivity and sporozoite intracellular calcium. Oocyst microcosms were sampled at two levels of insolation (T1 and T2) (see Table S1). **A**) Oocyst infectivity was determined using a cell culture TaqMan PCR infectivity assay for both dark and light irradiated oocysts. Non-irradiated oocysts kept at 4°C were used as controls, and treatments calculated as a percentage of the control. **B**) Oocysts were excysted and sporozoites incubated in supplemented medium at 37°C for 90 minutes before staining with the intracellular calcium indicator Fluo-4 AM and then analysed by flow cytometry. Sporozoite intracellular calcium is expressed as arbitrary units. An asterisk above a pair of bars indicate statistically significant effects (*t*-test, P<0.05). Error bars indicate standard deviations (n = 3).

*Cryptosporidium* oocysts exposed to solar UV were excysted and sporozoites stained with the calcium indicator Fluo-4 AM ester before flow cytometric analysis. Fluo-4 AM is a cell-permeant acetoxymethyl ester which can be loaded into cells. Non-specific esterases present in the cell then hydrolyze the AM ester, liberating the Ca^2+^ sensitive indicator. Upon binding intracellular calcium, the indictor exhibits a large increase in green fluorescence intensity [41]. Flow cytometric analysis of sporozoite cytosolic calcium identified a rise in calcium levels of sporozoites from solar irradiated oocysts in comparison to those excysted from dark controls (Fig. 6). A large reduction in oocyst infectivty for the treatments exposed to solar insolation was apparent in comparison to the dark controls only for the second sampling point T2 (Fig. 6A). A concurrent increase in sporozoite intracellular calcium was also evident at T2 (Fig. 6B). These changes were similar to changes previously reported in both intracellular calcium and internal granularity of excysted sporozoites treated with the ionophore A23187 and the depolarizing agent and inducer of exocytosis, potassium chloride [39]. The rapid release or influx of Ca^2+^ into the cytosol has been coupled to a number of key physiological processes including regulated exocytosis and apical organelle discharge and these processes have been previously shown to be intracellular calcium dependant for *C. parvum* [38].

To further confirm that the changes in sporozoite internal granularity, membrane depolarization, ATP and cytosolic calcium of solar irradiated oocysts were indeed reflective of more rapid sporozoite exocytosis, excysted sporozoites were stained with the exocytosis sensitive dye FM1-43 and exocytosed membrane quantified by flow cytometry and visualised by fluorescence microscopy. FM1-43 is a lipophilic styryl fluorescent dye. Upon binding to membranes, its quantum yield increases; however, it cannot cross from the outer to the inner leaflet of intact membranes and reversibility partitions into membranes [42]. These properties allow selective labelling of endosomes that form in the presence of dye. When secretory vesicles fuse with the surface membrane, the dye can diffuse through the fusion pore labelling newly exposed membrane. The resulting increase in fluorescence is a measure of the cumulative amount of membrane added by exocytosis [43].

**Figure 7:**
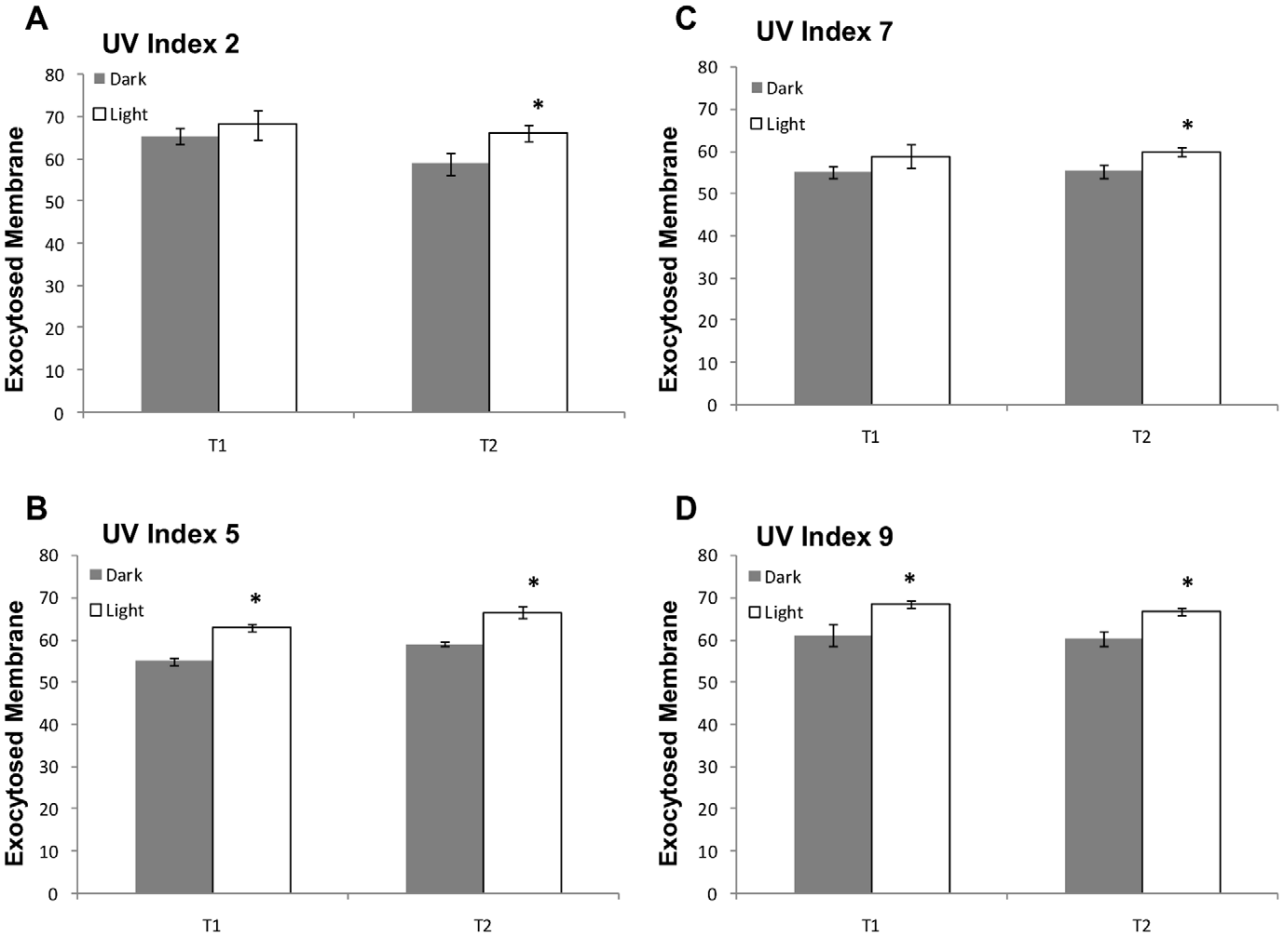
The effect of solar insolation on sporozoite exocytosis during multiple microcosm experiments. *Cryptosporidium* oocysts were exposed to solar radiation during four separate microcosm experiments (Table S1, microcosm experiments 2 and 4–6 (**A–D**) respectively). Oocyst microcosms were sampled at two levels of insolation (T1 and T2) for each microcosm experiment. The UV index for each experiment is presented at the top left hand corner of each graph. Oocysts were excysted and sporozoites stained with the exocytosis sensitive dye FM1-43 before incubation in supplemented medium at 37°C for 2.5 hour before flow cytometric analysis. Sporozoite exocytosed membrane is expressed as arbitrary units. An asterisk above a pair of bars indicate statistically significant effects (*t*-test, P<0.05). Error bars indicate standard deviations (n = 3). The infectivity data is presented in Figure S4.

Sporozoites excysted from solar irradiated oocysts consistently showed increased exocytosis in comparison to those of the dark controls (Fig. 7) however, it did not appear to be dosage dependent, with increased solar insolation (T2) not consistently resulting in augmented exocytosis. This is in contrast to strong dosage effects evident for both sporozoite membrane polarisation (Fig. S5) and internal granularity (Fig. S6). A possible explanation could be that the styryl dye FM1-43 may only provide a rough quantitative measurement of exocytosis in *Cryptosporidium* sporozoites as proteins and adhesions exocytosed are shed during helical gliding, along with possibly the dye. However, it must also be considered that membrane polarisation and sporozoite internal granularity are complex functions and while related to exocytosis may also be a function of other critical parameters affecting sporozoite infectivity including sporozoite membrane and vesicle integrity.

Fluorescence microscopy confirmed for those sporozoites subjected to solar UV, the distribution of the FM1-43 dye was predominately localised to the posterior end of the zoite as in the control treatments. The posterior distribution of exocytosed membrane was confirmatory of the motility of the sporozoites which undergo helical gliding as they advance upon a target cell [44]. Helical gliding is driven by coupling the translocation of surface adhesions to an actin-myosin motor beneath the parasite plasma membrane [45]. The surface-associated proteins, some which are present in the micronemes, are secreted from the anterior conoid of the zoite before translocation down the lateral membrane to the posterior [46]. Therefore, while sporozoites excysted from solar irradiated oocysts had begun to exocytose more rapidly, they were still capable of helical gliding and typical translocation of surface adhesions from the anterior to posterior region of the zoite. Helical gliding of solar irradiated sporozoites was further confirmed through fluorescence microscopy.

Apical discharge of secretory organelles has been demonstrated to be essential for attachment and invasion for a number of apicomplexans [18]. Proteins found in secretory organelles are known or hypothesised to be involved in host cell adhesion, parasitophorous sac formation and intracellular development, and the discharge of these organelles is intracellular calcium dependant [47]. While the host cell receptors and/or environmental cues found in the gastrointestinal tract that induce apical organelle discharge in the presence of a compatible cell are poorly understood for *C. parvum*, early initiation or inhibition of apical organelle discharge leads to reductions in cell invasion [38], [39].

**Figure 8:**
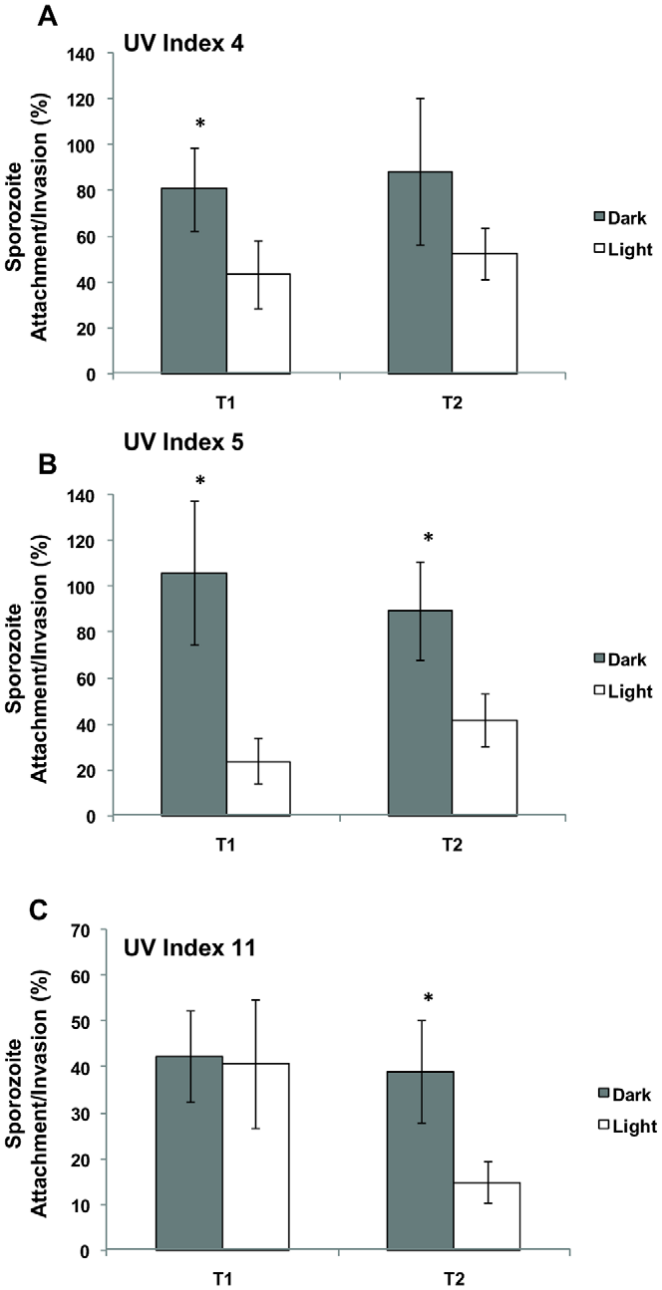
The effect of solar insolation on sporozoite attachment/invasion during multiple microcosm experiments. *Cryptosporidium* oocysts were exposed to solar radiation during three separate microcosm experiments (Table S1, microcosm experiments 3–4 and 7 (**A–C**) respectively). Oocyst microcosms were sampled at two levels of insolation (T1 and T2) for each microcosm experiment. The UV index for each experiment is presented at the top left hand corner of each graph. Sporozoite attachment/invasion was determined using a cell culture TaqMan PCR assay for both dark and light irradiated oocysts. Non-irradiated oocysts kept at 4°C were used as controls, and treatments calculated as a percentage of the control. An asterisk above a pair of bars indicate statistically significant effects (*t*-test, P<0.05). Error bars indicate standard deviations (n = 3). The infectivity data for **A** and **B** is presented in Figure S4 (**C** and **D**) and for **C**) in Figure 6.

We postulated that depolarization-dependant rises in cytoplasmic calcium induced by solar UV trigger premature secretion and subsequent shedding of proteins essential for the successful attachment and invasion of a compatible host cell, resulting in a false start to regulated exocytosis and consequently, a reduction in the ability of sporozoites to attach to/or invade a compatible host cell. To confirm this, an attachment/invasion assay was used to quantify the ability of sporozoites to attach or invade compatible target cells at 2 hours post infection. A reduction in the ability of sporozoites attaching to or invading cell monolayers was identified for those sporozoites excysted from solar irradiated oocysts (Fig. 8). For one time-point (Fig. 8C, T1) there was no reduction in infectivity/attachment, however this was matched by no significance decrease in infectivity (Fig. 6). With reductions exceeding 50 percent in a number of experiments, a significant driver behind *C. parvum*'s hypersensitivity to solar UV was apparent.

However, while sporozoite attachment/invasion was consistently less for solar irradiated oocysts in comparison to the dark controls, it did not appear to be dosage dependent. This is similar to the data generated from the exocytosis specific dye experiments and suggestive that while solar induced exocytosis may be sensitive to low levels of solar radiation, the relationship between increased irradiation and decreased cell attachment/invasion may not be linear; with the possibility solar induced exocytosis may plateau with increasing solar exposure.

Solar radiation induced exocytosis consequently appears to be an important contributor to the inhibition of *Cryptosporidium* infectivity. However, our inability to detect extensive DNA damage in *Cryptosporidium* sporozoites inactivated by solar radiation does not rule out DNA damage as a significant contributing factor to the inactivation of *Cryptosporidium*, especially considering that a considerable fraction of sporozoites were still able to attach *Cryptosporidium*. As *Cryptosporidium* merozoites do not form until 10–12 hours post infection, further infection time-course experiments may help resolve the contribution of these components by determining what percentage of solar UV irradiated sporozoites which are able to attach/invade remain viable until 10–12 hours post infection, where any interference in DNA replication would be expected to have dire consequences at this point in the infection cycle. Further to this, additional decreases in infectivity may be also driven by protein and/or membrane damage, while it is also possible that due to an exhaustion of finite energy reserves those sporozoites still able to attach perish before they can sequester the required metabolites from a compatible host cell.

Finally the UV components (UV-A/B) of solar insolation can vary considerably. The rationale behind the incorporation of the UV index and the use of the T_90_ value in the field design of these experiments was to a degree, to overcome this variation. However this design does not completely account for the effects and efficacy that different components of the UV spectrum may have on different cellular targets, which may explain some of the variation between and within experiments. Additional studies using long-pass filters may help resolve this matter.

### Conclusions

Our discovery that depolarization-dependant rises in cytoplasmic calcium induced by solar UV prematurely trigger exocytosis has significant implications not just for members of the Apicomplexa with a cyst lifecycle stage, but for a much wider group of organisms in environments experiencing increased levels of solar radiation. Throughout eukaryotic evolution, mechanisms of transport into and out of cells involving membrane fusion and fission have remained highly conserved [48]. While a variety of stimuli can elicit vesicular traffic, cell membrane depolarizations are a common trigger by means of voltage-operated channels and calcium induced calcium release mechanisms [49]. Disruption, modification or early elicitation of cellular secretion from increased exposure to solar UV may have unpredictable consequences for numerous organisms. Secondly, intracellular calcium plays a pivotal role as a second messenger for the control of a diverse variety of functions in eukaryotes, including contraction, cellular motility, cell division, differentiation, and ultimately cell death [49]. The versatility of calcium signalling allows the control of such a diverse range of processes however, exceeding the normal spatial and temporal boundaries of a cell can result in perturbed cellular function, including death. While intensive efforts have focused on the harmful effects of solar UV on an organism's DNA, perturbation of calcium signals by way of solar induced cell membrane depolarization may also have consequential effects for numerous organisms, aquatic and terrestrial, and require further attention.

## Materials and Methods

### Outdoor microcosm studies

An established experimental site in an unshaded area at Bolivar, 20 km north of Adelaide, 34° 55′S (latitude) 138° 36′E (longitude), South Australia was utilised for solar radiation experiments. Disposable sealable methylacrylate cuvettes (highly transmissible down to 285nm) were utilized as individual microcosms to house oocysts. Outdoor tanks constructed from 1m^3^ bulky bins and lined with heavy duty black pool plastic to reduce up-welling radiation were filled with approximately 1000 litres of tap water to act as a thermal jacket to reduce the effect of water temperature variation on oocysts housed inside cuvettes. Cuvettes were mounted on top of clear acrylic sheets which were weighted and submerged 10 cm below the surface within the tank. Temperature data loggers were placed at water depths corresponding to the submersed oocysts and temperatures recorded every 30 minutes. The temperature variation within experiments was no greater than 2°C. The temperature range of the water throughout the experimental periods was between 13°C–25°C, well within the temperature range shown to have a significant effect on oocyst infectivity [39], [40]. Cuvettes housing oocysts were wrapped in alfoil and submerged in tanks for dark controls. Cuvettes housing oocysts kept in the fridge at 4°C were used as controls to measure changes in the parameters quantified due to solar insolation and identify any effects on oocysts infectivity that were not resultant due to solar insolation. Each cuvette contained 200,000 oocysts, and individual cuvettes were sampled for each replicate from both dark and light treatments at each sampling point. All outdoor experiments commenced within 3 hours of solar noon.

### UV unit and UV-C dose calculations

A bench-scale collimated beam apparatus (Trojan Technologies Inc., Ontario, Canada) was used to irradiate oocysts for comparative studies of the effect of low pressure UV on DNA damage in comparison to the effect of outdoor insolation on DNA damage. This apparatus contained a low pressure 254nm mercury lamp. The sample to be irradiated at room temperature (approximately 22°C) was placed on a magnetic stir plate directly below the collimating tube (45cm). Irradiance was measured using a radiometer (International Light, Model 1L1400A, equipped with a 254nm UV detector model no. XRL140T254, Newburyport MA) calibrated to the standards of the US National Institute of Standards and Technology (NIST). The UV dose (mJ/cm^2^) was determined by multiplying the average irradiance (mW/cm^2^) in the sample liquid by the irradiation time (s). The low pressure UV doses were determined as previously described and calculated using software (Bolton Photosciences, Ayr, Canada) [50]. A petri factor, reflection factor and water factor were applied to all calculations. Oocyst stocks were prepared for collimated beam work at concentrations of 100,000 oocysts/ml with 5ml of oocyst stock used for each UV dose experiment.

### Immunoblot and Quantitative Sequence Detection (QSD) assays

After UV-C dose and outdoor microcosm experiments, irradiated oocysts were used for immunoblot and quantitative sequence detection (QSD) assays [28]. Briefly, for both immunoblot and QSD assays, crude DNA extractions were performed on 100,000 oocysts [30] exposed to a variety of UV-C dosages using a collimated beam (0–320mJ/cm^2^), or to varying levels of solar insolation in outdoor microcosm experiments. All extractions were performed in triplicate. For immunoblots, 5µl aliquots from a total extraction volume of 12µl were spotted and fixed to a Nylon Hybond^+^ membrane. For a single outdoor microcosm experiment, crude DNA extractions were also performed on 1 million oocysts for the immunoblot assay in an attempt to detect solar induced CPDs. UV-C induced damage was assessed through the use of a monoclonal antibody that recognized and bound specifically to cyclobutane pyrimidine dimers (mouse antibody H3) (1 in 4000 dilution). A secondary antibody labelled with an alkaline phosphatase conjugate targeted to the primary antibody was hybridised to the membrane (1 in 160,000 dilution) and visualised by incubation in a chemiluminescent substrate (CPD-star) and exposed to photographic film for varying levels of exposure. Plasmid DNA exposed to 360 mJ/cm^2^ of UV-C was quantified by a spectrophotometer and used as a standard (250pg-10ng). For QSD assays a 5µl aliquot was used in a total reaction volume of 25 µl using a previously described TaqMan assay [51]. Non-irradiated oocysts were used as controls, and the treatments calculated as a percentage of the control.

### Solar radiation measurements

Global (i.e., diffuse plus direct) solar radiation (GSR) was measured onsite by using a CM3 pyranometer connected to a Solrad Integrator data logger (Kipp and Zonen). Solar insolation measurements were taken at the beginning of all experiments and at each sampling point. The environmental UV index was recorded from the Australian Government Bureau of Meteorology website (www.bom.gov.au) during the experimental period and the type of day described as clear, broken cloud or overcast noted.

### Cell culture –TaqMan infectivity assays

*In vitro* culturing of the HCT-8 line (human ileocecal adenocarcinoma ATCC-CCL244, obtained from American Type Culture Collection) and *C. parvum* infection was undertaken as previously described [51]. Crude extraction of DNA from the infected monolayer and quantification of the level of cell culture infection was performed using Real-time PCR [8], [40]. Infectivity was calculated using 4°C incubated samples as controls using the equation: infectivity of the sample = (Taqman PCR results of the treatment/Taqman PCR result of the 4°C control), where the Taqman PCR result was the number of sporozoite equivalent bodies amplified in cell culture. For attachment/invasion assays, pre-treated oocysts were centrifuged onto the monolayer at 406 rcf for 5 minutes to increase the sensitivity of the assay. The parasite and cell recoveries were performed at 2 hours post infection of the HCT-8 cell line and quantified using the same TaqMan assay [51] and techniques described above.

### Flow cytometry

Flow cytometric quantification of sporozoite membrane depolarization, intracellular calcium and exocytosed membrane were performed using the vital dyes DiBAC4(3), Fluo-4 AM and FM1-43 respectively [39]. Oocyst excystation, fluorescent dye labelling and flow cytometric parameters were as reported therein. Excysted sporozoites to be with stained DiBAC4(3) and Fluo-4 AM were analysed by flow cytometry after 30 and 90 minutes incubation at 37°C respectively in supplemented RPMI medium post excystation treatment. Excysted sporozoites stained with FM1-43 were analysed by flow cytometry after 2.5 hours incubation at 37°C in supplemented RPMI medium post excystation treatment. A minimum of 20,000 events was collected for each treatment replicate of all the three dye stained particles. The region representing the sporozoites on the scatter plots was gated and histograms of fluorescence intensity plotted for the gated population. Sporozoites excysted from 4°C incubated controls were used as reference controls against dark and light treatments and histogram markers used to analyse the variation within the gated population and between treatments.

### ATP studies

Oocyst and sporozoite ATP levels were determined for solar radiation treatments using the ATPlite luminescence detection assay system using a luminescence counter (Wallac 1420 multilabel counter) to measure light emission [39], [40]. ATP standard curve construction and ATP extraction from oocysts and sporozoites was performed using a freeze/thaw lysis procedure previously described [39], [40].

### Microscopy

Excysted sporozoites stained with SYT09 and FM1-43 were also analysed by fluorescence microscopy. An Olympus BX60 microscope fitted with a 10× eyepiece and either a 40× or 100× objectives were used for examination of samples.

## Supporting Information

Figure S1 Quantification of the effect of solar insolation on Cyclobutane dimer formation within Cryptosporidium oocysts using a immunoblot assay. A microcosm experiment was performed over two consecutive clear sky days, both with a solar UV index maximum of 4 in order to investigate the formation of CPDs within oocysts. DNA extracts of oocysts exposed to a variety of solar insolation levels for both light and dark treatments were fixed to a Nylon Hybond+ membrane in triplicate. UV-C induced damage was assessed through the use of a monoclonal antibody that recognized and bound specifically to cyclobutane pyrimidine dimmers (CPDs). The chemiluminescent treated blot was exposed to photographic film for 40 minutes in an attempt to increase the level of detection. Plasmid DNA exposed to 360 mJ/cm2 of UV-C was used as a standard (250pg-10ng) and is located in the lower panel beneath the dashed line. CPDs were unable to be detected at any level of solar insolation in either the light or dark treatments.
(2.80 MB TIF)

Figure S2 Quantification of the effect of solar insolation on Cyclobutane dimer formation within Cryptosporidium oocysts using an immunoblot assay. In a further attempt to detect CPDs in solar irradiated oocysts, a crude DNA extraction was performed on 1 million oocysts exposed to 37,208kJ/m2 of solar insolation for both light and dark treatments. The outdoor microcosm experiment performed over two consecutive days, with solar UV index maxima of 4 (clear sky day) and 2 (cloudy day) respectively. DNA was fixed to a Nylon Hybond+ membrane in duplicate for both dark and light treatments. UV-C induced damage was assessed through the use of a monoclonal antibody that recognized and bound specifically to cyclobutane pyrimidine dimmers (CPDs). The chemiluminescent treated blot was exposed to photographic film for 45 minutes in an attempt to increase the level of detection. Plasmid DNA exposed to 360 mJ/cm2 of UV-C and used as a standard (250pg-10ng) is located in the upper panel above the dashed line. Dark controls demonstrated that the antibody did not detectably bind to oocyst DNA that had not been exposed to solar insolation. CPDs were able to be detected at this level of solar insolation in the light treatments by increasing quantity of oocysts DNA. Oocyst cell culture inactivation data for this work is presented in Figure 3D.
(1.78 MB TIF)

Figure S3 Calculation of expected T90 values for days of varying solar UV indices. A single plot defining the relationship between UV index and the Cryptosporidium oocyst T90 value (the time taken to achieve a 90% reduction in cell culture infectivity as determined by the cell culture-TaqMan assay) in Bolivar tap water. Each T90 value was derived from an individual outdoor microcosm experiment of infectivity vs time (hours of exposure) over multiple solar insolation exposures [8].
(0.11 MB TIF)

Figure S4 The effect of solar insolation on Cryptosporidium oocyst infectivity during multiple microcosm experiments. Oocysts were exposed to solar radiation during six separate microcosm experiments (Table 1S, microcosm experiments 1–6, (A–F) respectively). Oocyst microcosms were sampled at two levels of insolation (T1 and T2) for each microcosm experiment. Oocyst infectivity was determined using a cell culture TaqMan PCR infectivity assay for both dark and light irradiated oocysts. Non-irradiated oocysts kept at 4°C were used as controls and treatments calculated as a percentage of the control. An asterisk above a pair of bars indicate statistically significant effects (t-test, P<0.05). Error bars indicate standard deviations (n = 3).
(0.22 MB TIF)

Figure S5 The effect of solar insolation on Cryptosporidium sporozoite membrane polarization during multiple microcosm experiments. Oocysts were exposed to solar radiation during six separate microcosm experiments (Table S1, microcosm experiments 1–6, (A–F) respectively). Oocyst microcosms were sampled at two levels of insolation (T1 and T2) for each microcosm experiment. Oocysts were excysted and sporozoites incubated for 30 minutes in supplemented medium at 37°C before staining with the membrane potential sensitive dye DiBAC4(3) and subsequent flow cytometric analysis. The gated sporozoite population was analysed on the FL-1 channel. Non-irradiated oocysts kept at 4°C were used as controls and treatments calculated as a percentage of the control. An asterisk above a pair of bars indicate statistically significant effects (t-test, P<0.05). Error bars indicate standard deviations (n = 3).
(0.26 MB TIF)

Figure S6 The effect of solar insolation on Cryptosporidium sporozoite internal granularity during multiple microcosm experiments. Oocysts were exposed to solar radiation during six separate microcosm experiments (Table S1, microcosm experiments 1–6, (A–F) respectively). Oocyst microcosms were sampled at two levels of insolation (T1 and T2) for each microcosm experiment. Oocysts were excysted and sporozoites incubated for 30 minutes in supplemented medium at 37°C before staining with the membrane potential sensitive dye DiBAC4(3) and subsequent flow cytometric analysis. The gated sporozoite population was analysed on the SSC channel. Non-irradiated oocysts kept at 4°C were used as controls and treatments calculated as a percentage of the control. An asterisk above a pair of bars indicate statistically significant effects (t-test, P<0.05). Error bars indicate standard deviations (n = 3).
(0.27 MB TIF)

Figure S7 The effect of solar insolation on oocyst infectivity, sporozoite membrane polarization and internal granularity during a single microcosm experiment. Cryptosporidium oocysts exposed to solar radiation during a single microcosm experiment on a clear sky day with a UV index maximum of 3 were sampled at increasing levels of solar insolation during the course of the experiment. A) Oocyst infectivity was determined using a cell culture TaqMan PCR infectivity assay for both dark and light irradiated oocysts. Non-irradiated oocysts kept at 4°C were used as controls and treatments calculated as a percentage of the control. Oocysts sampled at the same time-points corresponding to each insolation level were excysted and sporozoites stained with the membrane potential sensitive dye DiBAC4(3) before flow cytometric analysis on the FL-1 (B) and side scatter channels (C). Both sporozoite membrane polarization and internal granularity are expressed as a percentage of the non-irradiated oocyst controls. D) Strong correlations were established between reductions in oocyst infectivity and sporozoite membrane potential, E) as well as between oocyst infectivity and sporozoite internal granularity. Error bars indicate standard deviations for infectivity (n = 3).
(0.23 MB TIF)

Figure S8 The effect of solar insolation on excysted sporozoite ATP content during three separate microcosm experiments. Cryptosporidium oocysts were exposed to solar radiation during three separate microcosm experiments (Table S1, microcosm experiments 3–5, (A–C) respectively). Oocyst microcosms were sampled at two levels of insolation (T1 and T2) for each microcosm experiment. Oocysts were excysted and sporozoites incubated for 30 minutes in supplemented medium at 37°C before ATP extraction and analysis. An asterisk above a pair of bars indicate statistically significant effects (t-test, P<0.05). Error bars indicate standard deviations (n = 3). The infectivity data is presented in Figure S7.
(0.17 MB TIF)

Figure S9 The effect of solar insolation on oocyst infectivity and ATP content during a single microcosm experiment. Cryptosporidium oocysts were exposed to solar radiation during a single microcosm experiment performed over three consecutive days, with solar UV index maxima of 1 (cloudy), 1 (cloudy), and 2 (clear sky) respectively. Oocysts were sampled at increasing levels of solar insolation during the course of the experiment. A) Oocyst infectivity was determined using a cell culture TaqMan PCR infectivity assay for both dark and light irradiated oocysts. Non-irradiated oocysts kept at 4°C were used as controls and treatments calculated as a percentage of the control. Oocysts were sampled at the same time-points corresponding to each insolation level and ATP extractions undertaken. B) ATP assays were performed on oocysts immediately after solar irradiation treatments. Oocysts sampled at the same insolation levels were also incubated at 37°C for C) 8 hours and D) 24 hours holding periods before oocyst ATP extraction and analysis. An asterisk above a pair of bars indicate statistically significant effects (t-test, P<0.05). Error bars indicate standard deviations (n = 3).
(0.26 MB TIF)

Table S1 UV Index, solar insolation levels and temperature of the outdoor solar inactivation microcosm experiments undertaken for investigation of the effect of solar insolation on sporozoite membrane potential, granularity and infectivity as presented in Figure 4 and Figures S4, S5, S6. Sporozoite ATP and sporozoite exocytosis were quantified in a number of these experiments and the results presented in Figures S8 and 7 respectively.
(0.35 MB TIF)
