# Supplementary figures and images for: Solar Radiation Induces Non-Nuclear Perturbations and a False Start to Regulated Exocytosis in Cryptosporidium parvum
Source: PLoS One. 2010 Jul 23;5(7):e11773. doi: 10.1371/journal.pone.0011773 (PMC2909268; doi:10.1371/journal.pone.0011773)

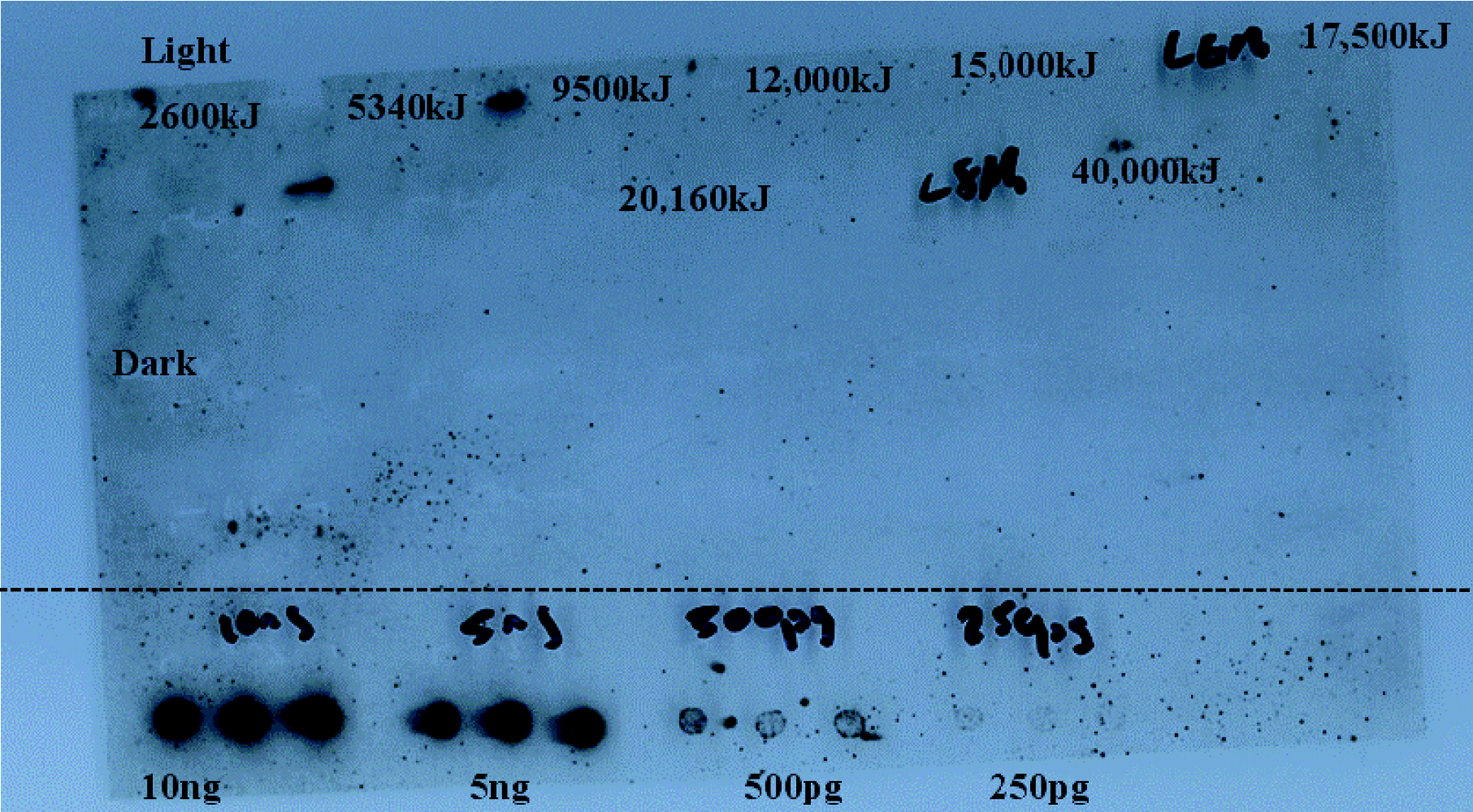

Supplement: Figure S1 — Quantification of the effect of solar insolation on Cyclobutane dimer formation within Cryptosporidium oocysts using a immunoblot assay. A microcosm experiment was performed over two consecutive clear sky days, both with a solar UV index maximum of 4 in order to investigate the formation of CPDs within oocysts. DNA extracts of oocysts exposed to a variety of solar insolation levels for both light and dark treatments were fixed to a Nylon Hybond+ membrane in triplicate. UV-C induced damage was assessed through the use of a monoclonal antibody that recognized and bound specifically to cyclobutane pyrimidine dimmers (CPDs). The chemiluminescent treated blot was exposed to photographic film for 40 minutes in an attempt to increase the level of detection. Plasmid DNA exposed to 360 mJ/cm2 of UV-C was used as a standard (250pg-10ng) and is located in the lower panel beneath the dashed line. CPDs were unable to be detected at any level of solar insolation in either the light or dark treatments. (2.80 MB TIF) [file pone.0011773.s001.tif]

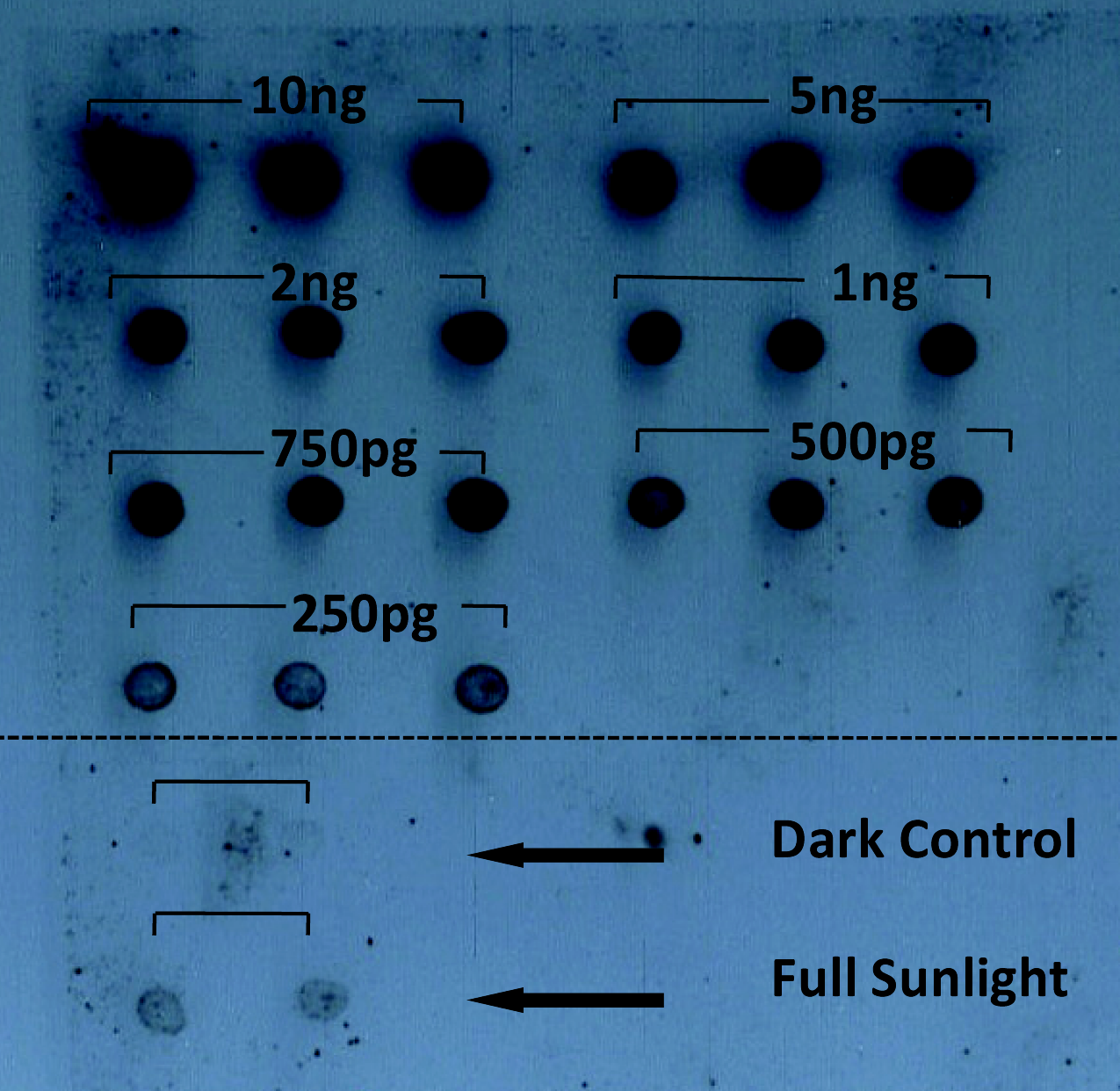

Supplement: Figure S2 — Quantification of the effect of solar insolation on Cyclobutane dimer formation within Cryptosporidium oocysts using an immunoblot assay. In a further attempt to detect CPDs in solar irradiated oocysts, a crude DNA extraction was performed on 1 million oocysts exposed to 37,208kJ/m2 of solar insolation for both light and dark treatments. The outdoor microcosm experiment performed over two consecutive days, with solar UV index maxima of 4 (clear sky day) and 2 (cloudy day) respectively. DNA was fixed to a Nylon Hybond+ membrane in duplicate for both dark and light treatments. UV-C induced damage was assessed through the use of a monoclonal antibody that recognized and bound specifically to cyclobutane pyrimidine dimmers (CPDs). The chemiluminescent treated blot was exposed to photographic film for 45 minutes in an attempt to increase the level of detection. Plasmid DNA exposed to 360 mJ/cm2 of UV-C and used as a standard (250pg-10ng) is located in the upper panel above the dashed line. Dark controls demonstrated that the antibody did not detectably bind to oocyst DNA that had not been exposed to solar insolation. CPDs were able to be detected at this level of solar insolation in the light treatments by increasing quantity of oocysts DNA. Oocyst cell culture inactivation data for this work is presented in Figure 3D. (1.78 MB TIF) [file pone.0011773.s002.tif]

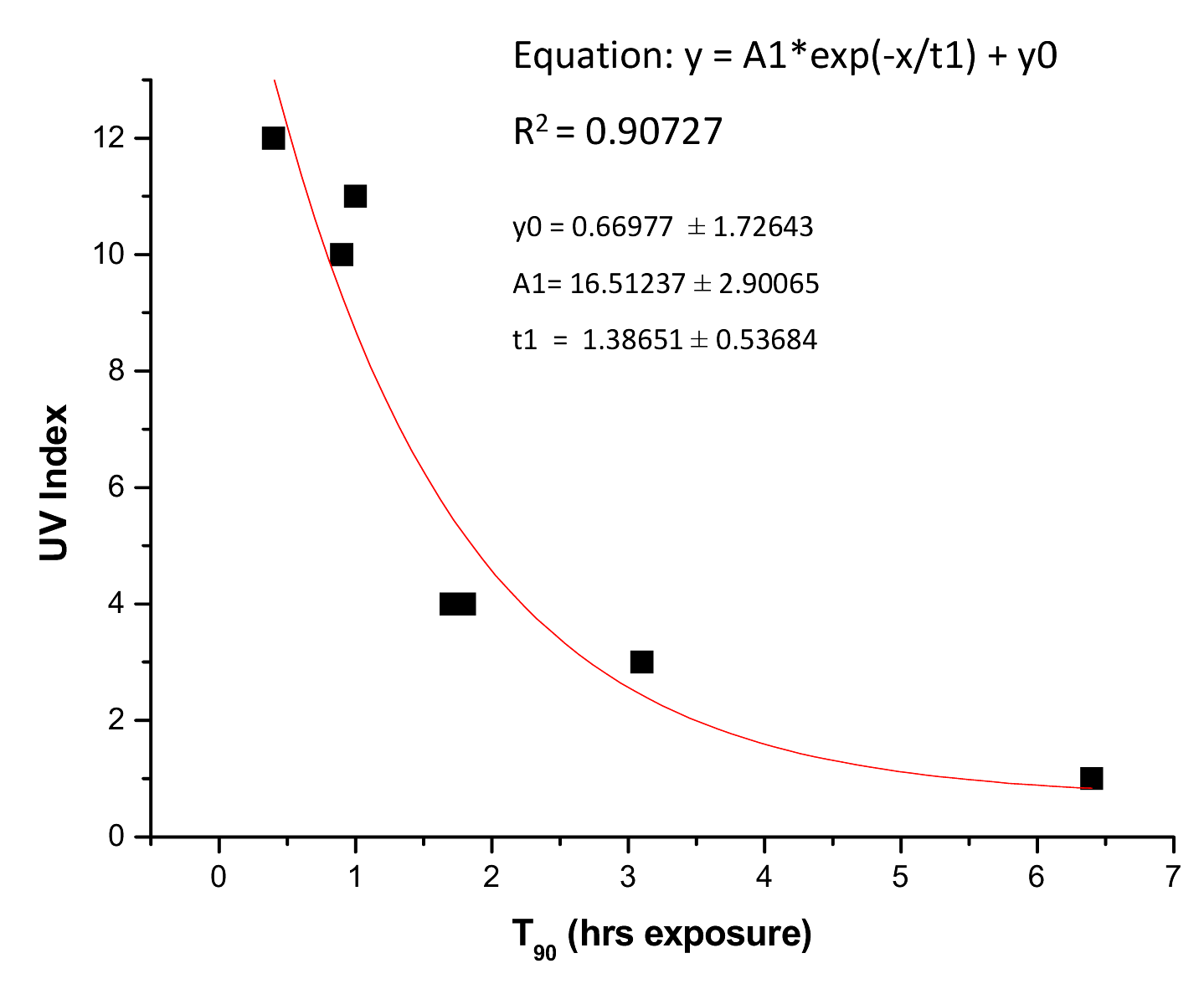

Supplement: Figure S3 — Calculation of expected T90 values for days of varying solar UV indices. A single plot defining the relationship between UV index and the Cryptosporidium oocyst T90 value (the time taken to achieve a 90% reduction in cell culture infectivity as determined by the cell culture-TaqMan assay) in Bolivar tap water. Each T90 value was derived from an individual outdoor microcosm experiment of infectivity vs time (hours of exposure) over multiple solar insolation exposures [8]. (0.11 MB TIF) [file pone.0011773.s003.tif]

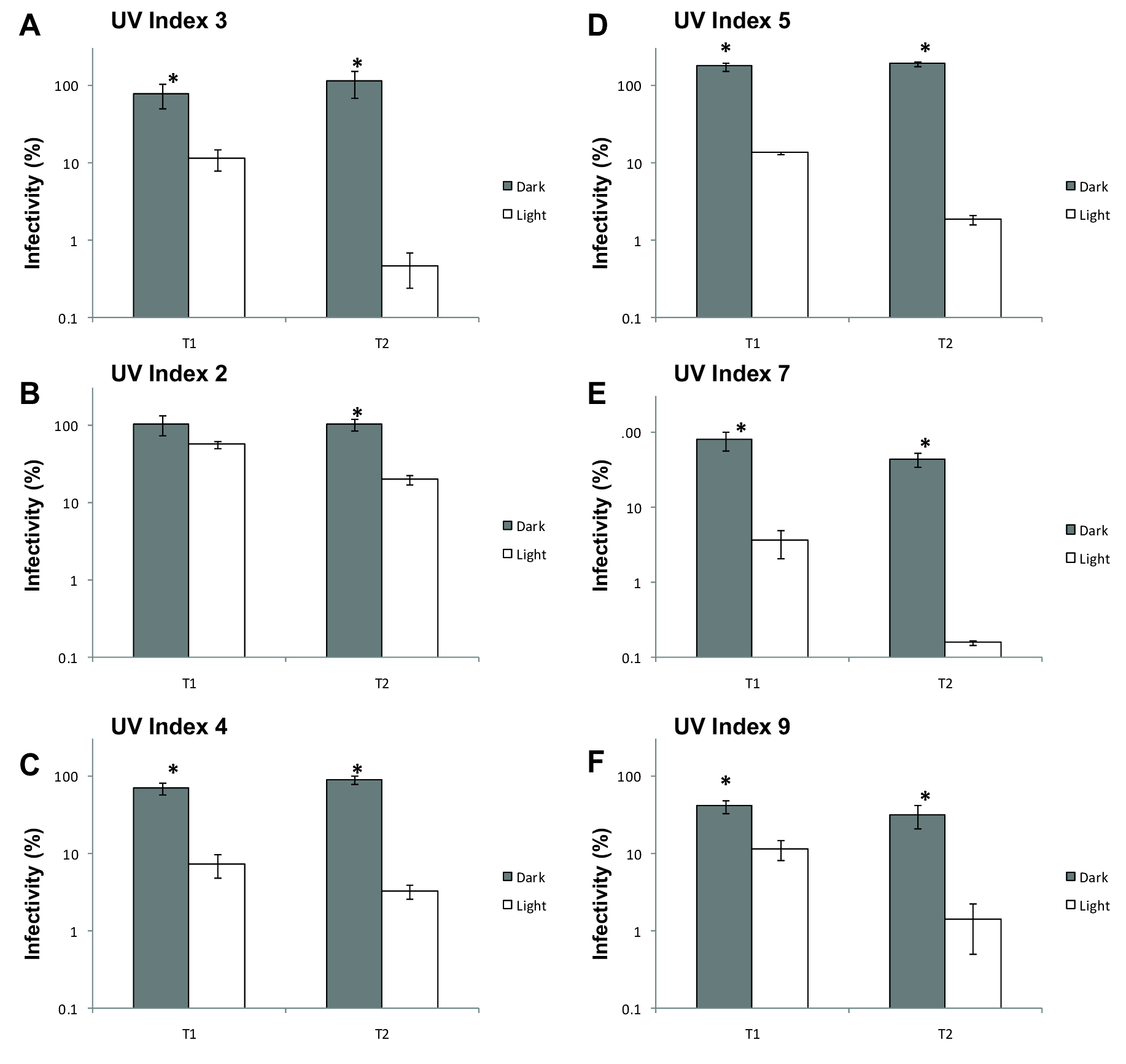

Supplement: Figure S4 — The effect of solar insolation on Cryptosporidium oocyst infectivity during multiple microcosm experiments. Oocysts were exposed to solar radiation during six separate microcosm experiments (Table 1S, microcosm experiments 1–6, (A–F) respectively). Oocyst microcosms were sampled at two levels of insolation (T1 and T2) for each microcosm experiment. Oocyst infectivity was determined using a cell culture TaqMan PCR infectivity assay for both dark and light irradiated oocysts. Non-irradiated oocysts kept at 4°C were used as controls and treatments calculated as a percentage of the control. An asterisk above a pair of bars indicate statistically significant effects (t-test, P<0.05). Error bars indicate standard deviations (n = 3). (0.22 MB TIF) [file pone.0011773.s004.tif]

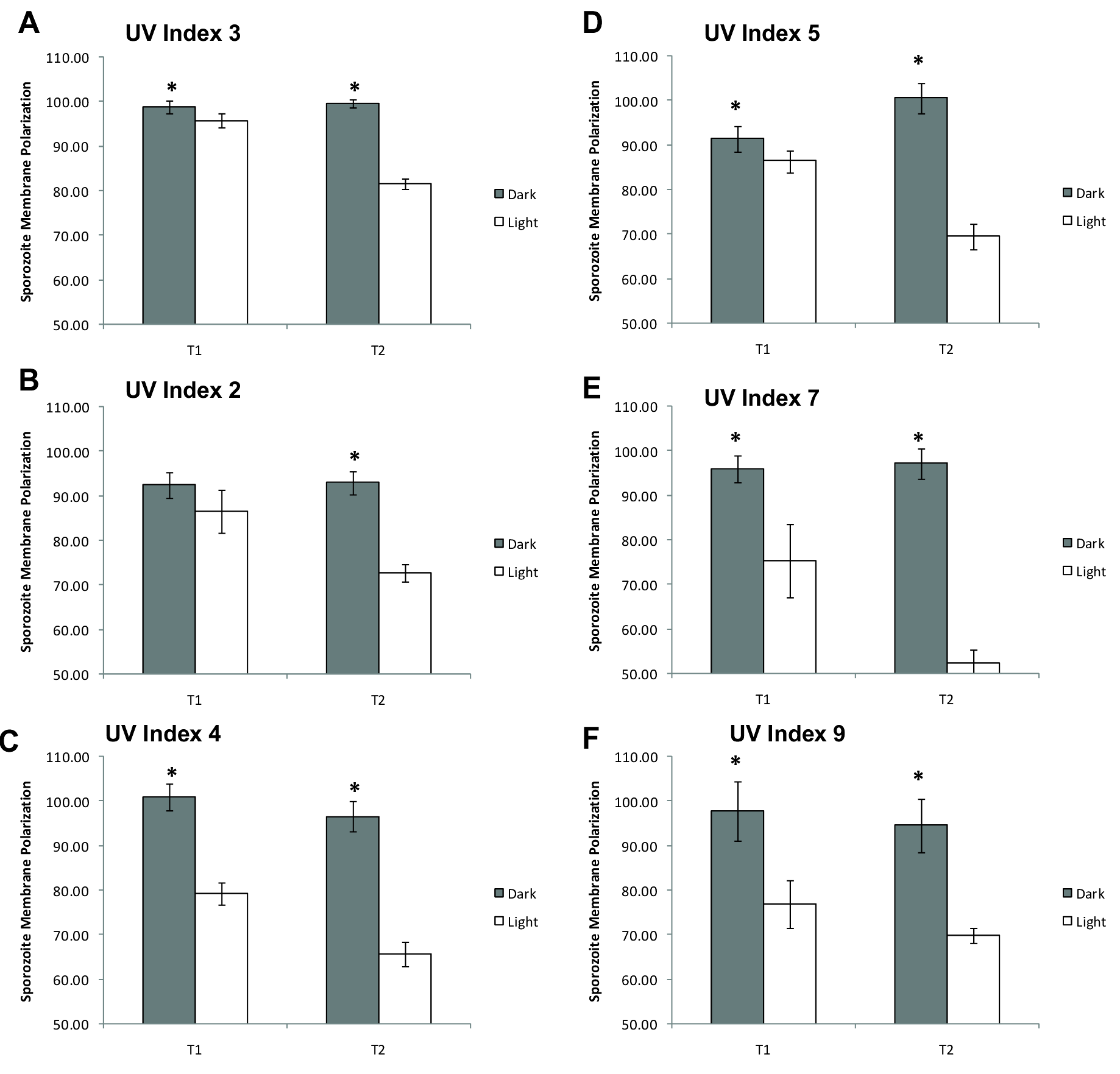

Supplement: Figure S5 — The effect of solar insolation on Cryptosporidium sporozoite membrane polarization during multiple microcosm experiments. Oocysts were exposed to solar radiation during six separate microcosm experiments (Table S1, microcosm experiments 1–6, (A–F) respectively). Oocyst microcosms were sampled at two levels of insolation (T1 and T2) for each microcosm experiment. Oocysts were excysted and sporozoites incubated for 30 minutes in supplemented medium at 37°C before staining with the membrane potential sensitive dye DiBAC4(3) and subsequent flow cytometric analysis. The gated sporozoite population was analysed on the FL-1 channel. Non-irradiated oocysts kept at 4°C were used as controls and treatments calculated as a percentage of the control. An asterisk above a pair of bars indicate statistically significant effects (t-test, P<0.05). Error bars indicate standard deviations (n = 3). (0.26 MB TIF) [file pone.0011773.s005.tif]

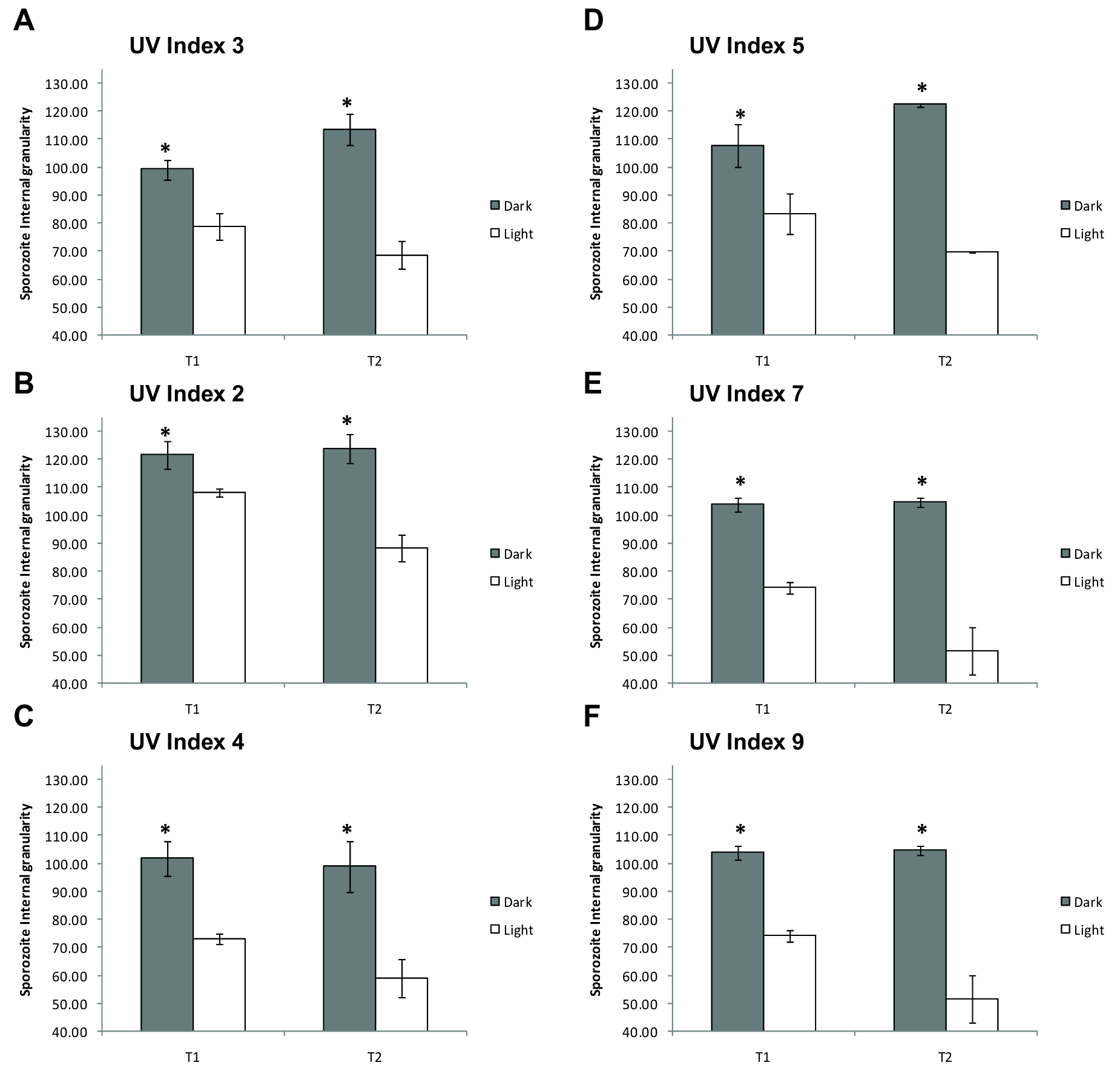

Supplement: Figure S6 — The effect of solar insolation on Cryptosporidium sporozoite internal granularity during multiple microcosm experiments. Oocysts were exposed to solar radiation during six separate microcosm experiments (Table S1, microcosm experiments 1–6, (A–F) respectively). Oocyst microcosms were sampled at two levels of insolation (T1 and T2) for each microcosm experiment. Oocysts were excysted and sporozoites incubated for 30 minutes in supplemented medium at 37°C before staining with the membrane potential sensitive dye DiBAC4(3) and subsequent flow cytometric analysis. The gated sporozoite population was analysed on the SSC channel. Non-irradiated oocysts kept at 4°C were used as controls and treatments calculated as a percentage of the control. An asterisk above a pair of bars indicate statistically significant effects (t-test, P<0.05). Error bars indicate standard deviations (n = 3). (0.27 MB TIF) [file pone.0011773.s006.tif]

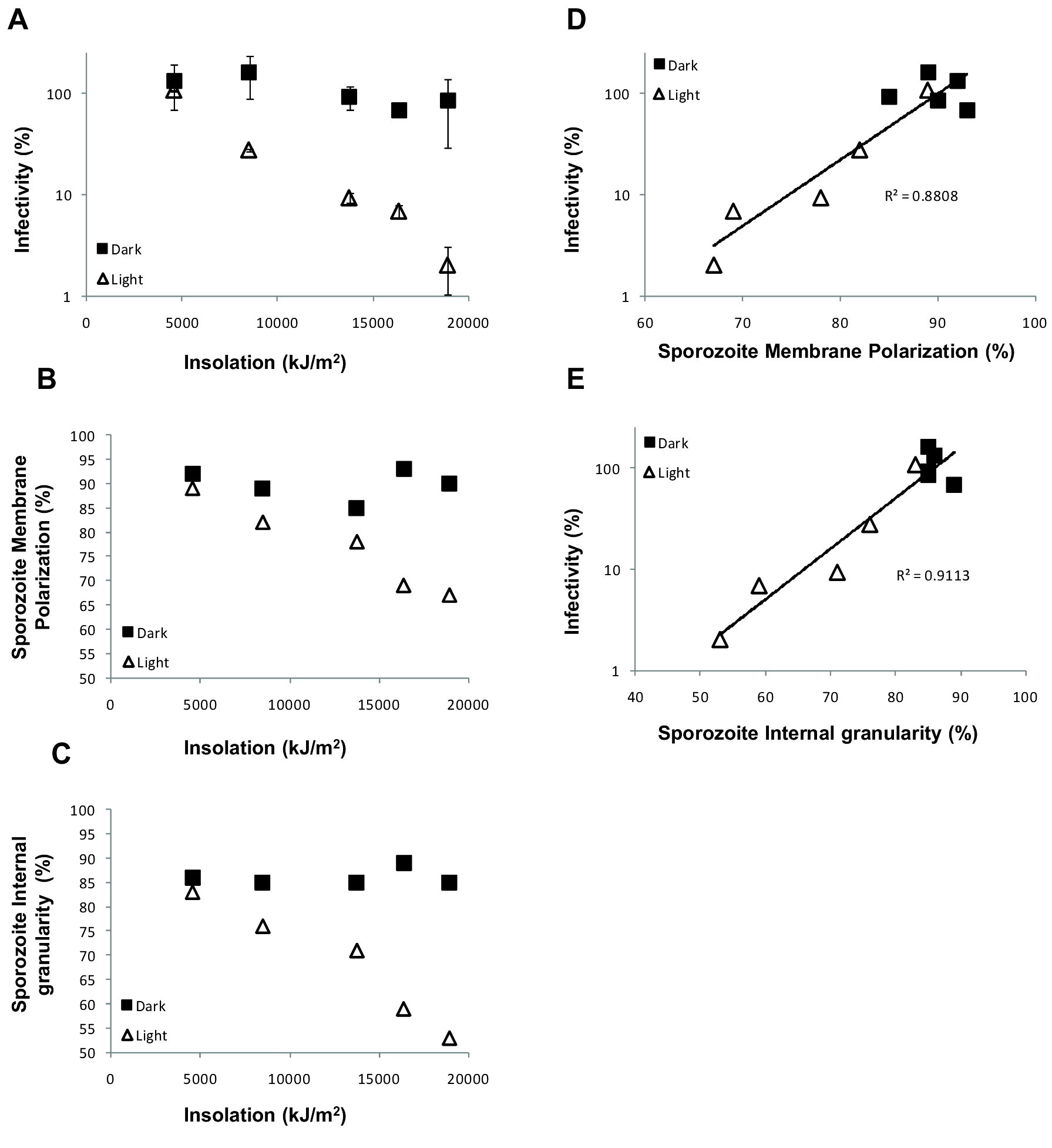

Supplement: Figure S7 — The effect of solar insolation on oocyst infectivity, sporozoite membrane polarization and internal granularity during a single microcosm experiment. Cryptosporidium oocysts exposed to solar radiation during a single microcosm experiment on a clear sky day with a UV index maximum of 3 were sampled at increasing levels of solar insolation during the course of the experiment. A) Oocyst infectivity was determined using a cell culture TaqMan PCR infectivity assay for both dark and light irradiated oocysts. Non-irradiated oocysts kept at 4°C were used as controls and treatments calculated as a percentage of the control. Oocysts sampled at the same time-points corresponding to each insolation level were excysted and sporozoites stained with the membrane potential sensitive dye DiBAC4(3) before flow cytometric analysis on the FL-1 (B) and side scatter channels (C). Both sporozoite membrane polarization and internal granularity are expressed as a percentage of the non-irradiated oocyst controls. D) Strong correlations were established between reductions in oocyst infectivity and sporozoite membrane potential, E) as well as between oocyst infectivity and sporozoite internal granularity. Error bars indicate standard deviations for infectivity (n = 3). (0.23 MB TIF) [file pone.0011773.s007.tif]

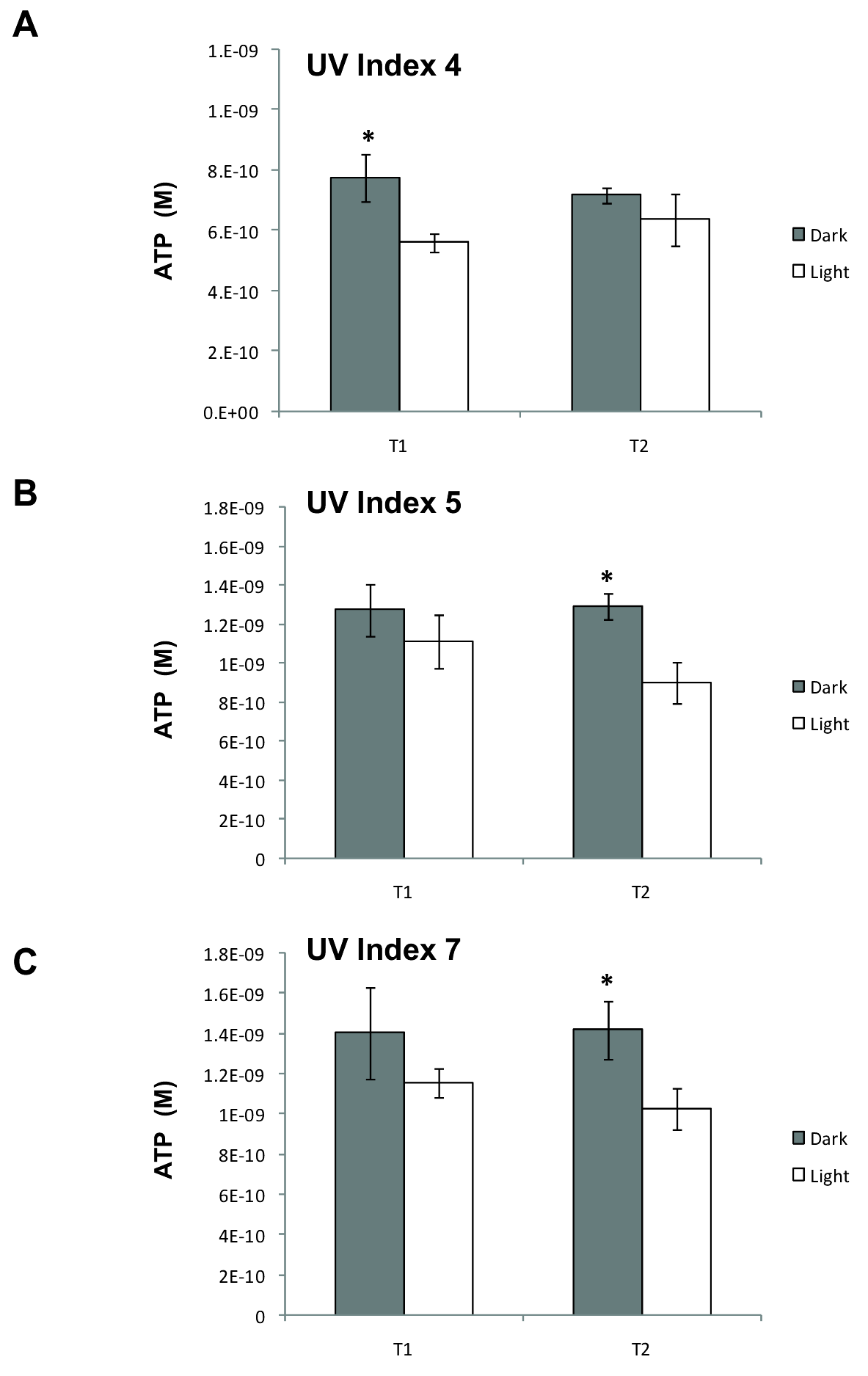

Supplement: Figure S8 — The effect of solar insolation on excysted sporozoite ATP content during three separate microcosm experiments. Cryptosporidium oocysts were exposed to solar radiation during three separate microcosm experiments (Table S1, microcosm experiments 3–5, (A–C) respectively). Oocyst microcosms were sampled at two levels of insolation (T1 and T2) for each microcosm experiment. Oocysts were excysted and sporozoites incubated for 30 minutes in supplemented medium at 37°C before ATP extraction and analysis. An asterisk above a pair of bars indicate statistically significant effects (t-test, P<0.05). Error bars indicate standard deviations (n = 3). The infectivity data is presented in Figure S7. (0.17 MB TIF) [file pone.0011773.s008.tif]

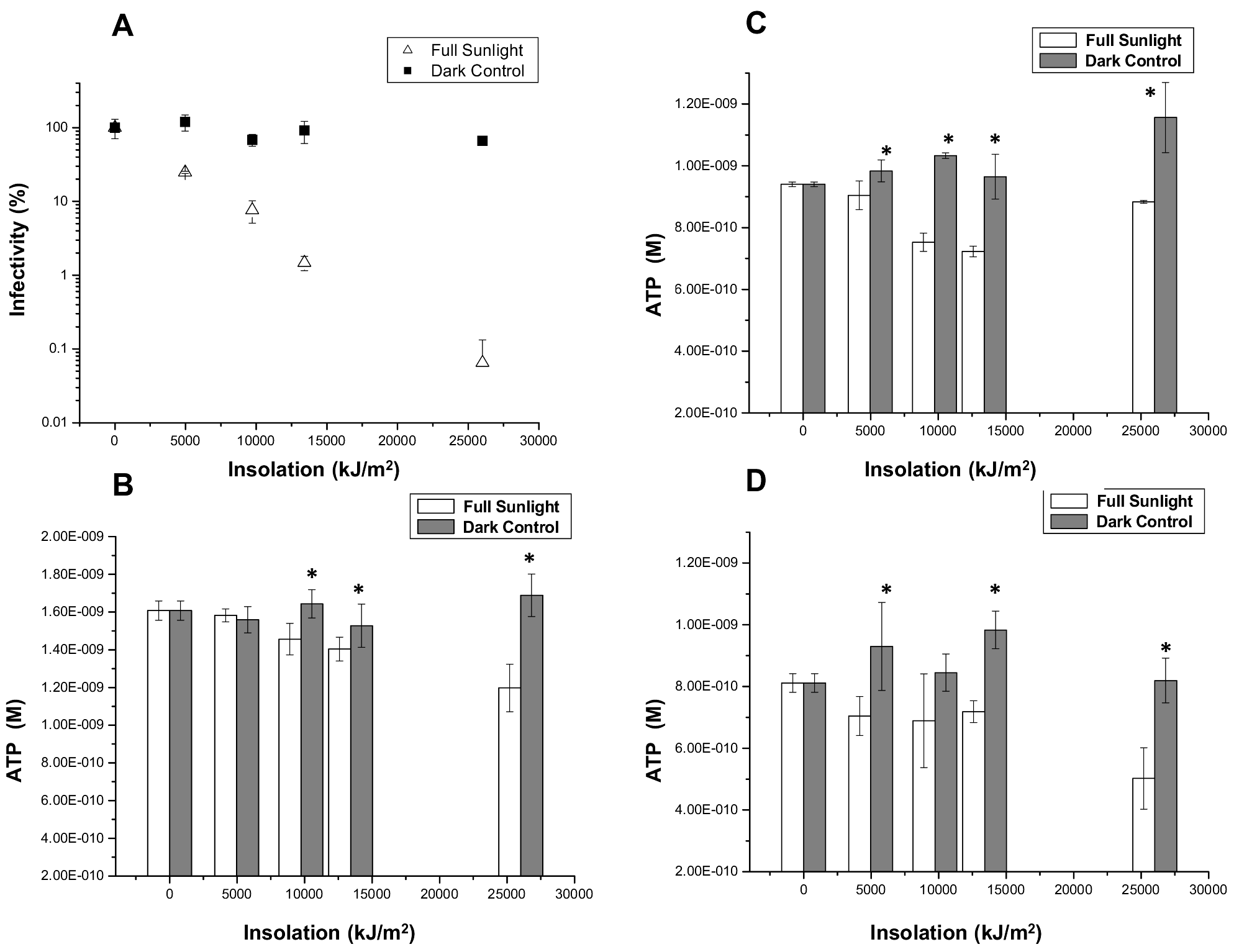

Supplement: Figure S9 — The effect of solar insolation on oocyst infectivity and ATP content during a single microcosm experiment. Cryptosporidium oocysts were exposed to solar radiation during a single microcosm experiment performed over three consecutive days, with solar UV index maxima of 1 (cloudy), 1 (cloudy), and 2 (clear sky) respectively. Oocysts were sampled at increasing levels of solar insolation during the course of the experiment. A) Oocyst infectivity was determined using a cell culture TaqMan PCR infectivity assay for both dark and light irradiated oocysts. Non-irradiated oocysts kept at 4°C were used as controls and treatments calculated as a percentage of the control. Oocysts were sampled at the same time-points corresponding to each insolation level and ATP extractions undertaken. B) ATP assays were performed on oocysts immediately after solar irradiation treatments. Oocysts sampled at the same insolation levels were also incubated at 37°C for C) 8 hours and D) 24 hours holding periods before oocyst ATP extraction and analysis. An asterisk above a pair of bars indicate statistically significant effects (t-test, P<0.05). Error bars indicate standard deviations (n = 3). (0.26 MB TIF) [file pone.0011773.s009.tif]

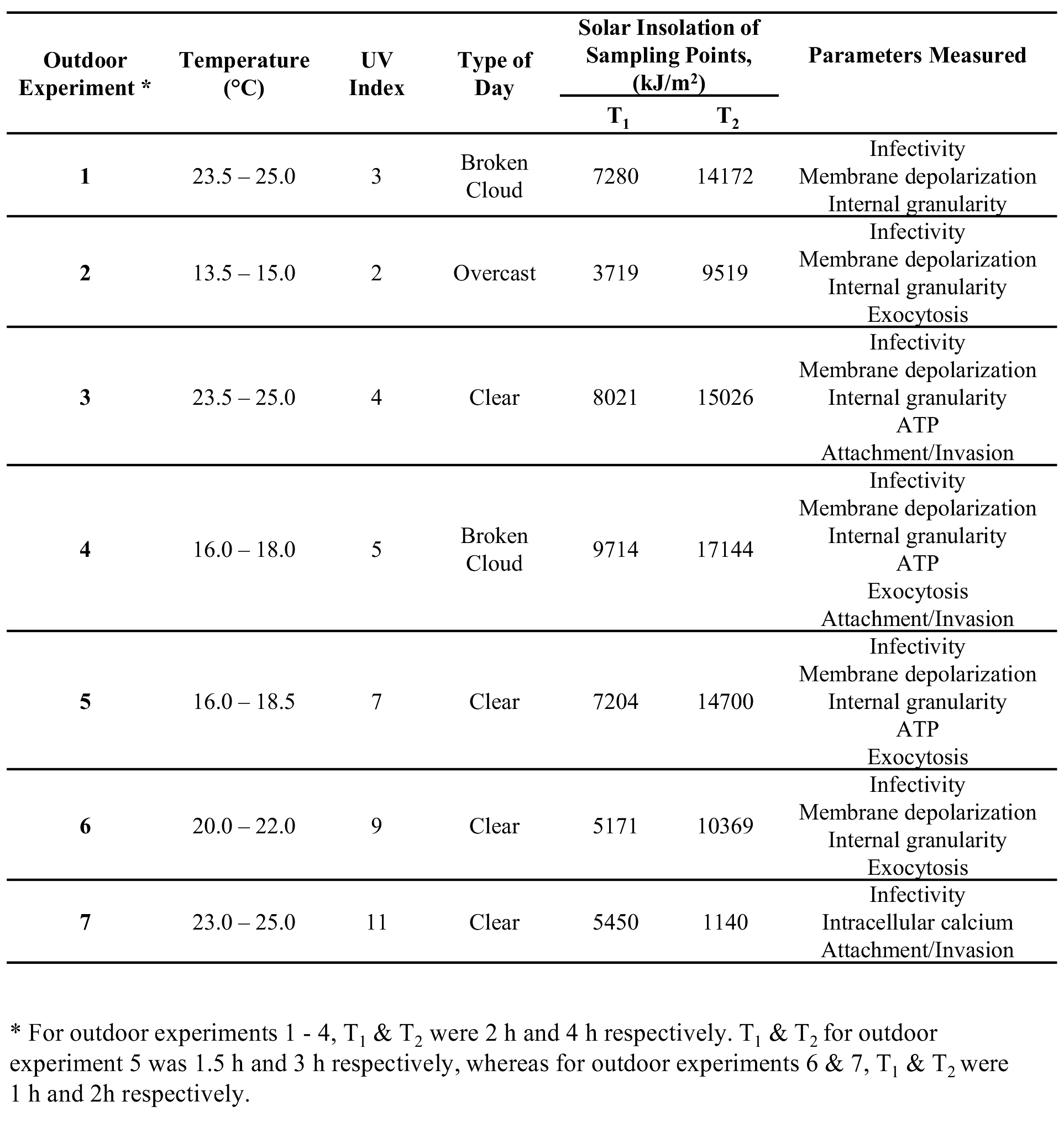

Supplement: Table S1 — UV Index, solar insolation levels and temperature of the outdoor solar inactivation microcosm experiments undertaken for investigation of the effect of solar insolation on sporozoite membrane potential, granularity and infectivity as presented in Figure 4 and Figures S4, S5, S6. Sporozoite ATP and sporozoite exocytosis were quantified in a number of these experiments and the results presented in Figures S8 and 7 respectively. (0.35 MB TIF) [file pone.0011773.s010.tif]
